# Handling missing items in the Hospital Anxiety and Depression Scale (HADS): a simulation study

**DOI:** 10.1186/s13104-016-2284-z

**Published:** 2016-10-22

**Authors:** Melanie L. Bell, Diane L. Fairclough, Mallorie H. Fiero, Phyllis N. Butow

**Affiliations:** 1 Department of Epidemiology and Biostatistics, Mel and Enid Zuckerman College of Public Health, University of Arizona, 1295N. Martin Ave., P.O. Box 245163, Tucson, AZ 85724 USA; 2 Department of Biostatistics and Informatics, Colorado School of Public Health, 13001 E. 17th Place, Campus Box B119, Aurora, CO 80045 USA; 3 Psycho-Oncology Co-Operative Research Group, School of Psychology, The University of Sydney, Level 6-North, The Lifehouse, 119-143 Missenden Rd, Sydney, NSW 2006 Australia

**Keywords:** Missing data, Imputation, Questionnaires, Simulation, Anxiety, Depression, Distress

## Abstract

**Background:**

The Hospital Anxiety and Depression Scale (HADS) is a widely used questionnaire in health research, but there is little guidance on how to handle missing items. We aimed to investigate approaches to handling item non-response, varying sample size, proportion of subjects with missing items, proportion of missing items per subject, and the missingness mechanism.

**Methods:**

We performed a simulation study based on anxiety and depression data among cancer survivors and patients. Item level data were deleted according to random, demographic, and subscale dependent missingness mechanisms. Seven methods for handling missing items were assessed for bias and imprecision. Imputation, imputation conditional on the number of non-missing items, and complete case approaches were used. One thousand datasets were simulated for each parameter combination.

**Results:**

All methods were most sensitive when missingness was dependent on the subscale (i.e., higher values of depression leads to higher levels of missingness). The worst performing approach was to analyze only individuals with complete data. The best performing imputation methods depended on whether inference was targeted at the individual or at the population.

**Conclusions:**

We recommend the ‘half rule’ using individual subscale means when using the HADS scores at the individual level (e.g. screening). For population inference, we recommend relaxing the requirement that at least half the items be answered to minimize missing scores.

**Electronic supplementary material:**

The online version of this article (doi:10.1186/s13104-016-2284-z) contains supplementary material, which is available to authorized users.

## Background

The Hospital Anxiety and Depression Scale (HADS) [1] is a widely used questionnaire in health research. A 14-item questionnaire with two subscales, researchers have used the sub-scales separately, or as a composite score to measure or screen for distress, in various fields including oncology, cardiology, psychology and psychiatry, both in research and clinical capacities [2]. It has been shown to be valid and reliable in a variety of settings [3]. Despite its widespread use, and multiple investigations into its validity [4], there are no guidelines for how to handle missing items and users must make ad-hoc decisions about what to do about missing items.

Missing data is ubiquitous in human research, both in randomized trials and observational studies; whether the design is longitudinal or cross-sectional. In longitudinal designs research participants may be lost to follow-up or may intermittently skip assessments, so that their entire questionnaires are missing. In both longitudinal and cross sectional designs participants may skip individual items on questionnaires. Both of these types of missingness have two possible implications: (1) reduced sample size and therefore lower power and (2) bias, if the missingness is non-random [5]. It is difficult to know exactly how researchers handle missing HADS items in practice. The most common approach for missing outcomes in RCTs, however, is complete case analysis, i.e., discarding data which are not complete [6]. If this is true for item level missingness, researchers are at risk of bias and imprecision in estimation, depending on the amount of missing item data.

If an item is missing, the entire subscale or questionnaire could be deemed missing, a method sometimes called case deletion or complete case. This has the effect of reducing sample size. If items are missing randomly, for example, because a subject did not see the item and therefore did not answer it, only power is affected. If items are missing non-randomly, however, excluding the subject’s entire score is likely to result in bias. For example, if subjects who are more anxious are less likely to answer all the questions, and a case deletion rule was used, anxiety could be underestimated. This is an example of *subscale dependent missingness.* Missingness may also depend on other factors, such as subject characteristics like demographics, risk factors, or health variables (for example, if men are more likely to have non-missing items than women). This is an example of *demographic dependent missingness.* In addition to case deletion, filling in missing item values, or imputation, is another missing item approach. Imputing missing items may take care of both power and bias issues, but there are several possible imputation methods, as detailed below, and the best one for the HADS has not been determined.

The lack of guidance on how to handle missing items in the HADS is in contrast to two well-known questionnaires, the Functional Assessment of Cancer Therapy General (FACT-G) which measures quality of life for cancer patients and the SF36, which measures wellbeing in the general population. The recommended method for these questionnaires for missing items is to replace the missing items with the mean of the answered items in the subscale, if at least half of that subscale has been answered [7–9]. This is sometimes called the half-rule, and is appealing because it is simple, is not sample dependent and can be performed at the time of questionnaire scoring. The rationale behind the half-rule is that an individual’s score would not have enough information to be valid if fewer items than half were answered. It is unknown how most HADS users handle item non-response. Jörngården et al. use the half-rule; an education and health psychology company’s website states that a mean imputation may be used, but only in the case of a single missing item (if more than one item is missing they state that the subscale is invalid). Multiple imputation is an approach that has been investigated and found to have good properties [10, 11], although implementation for outcomes research can be challenging [10, 12], and it could not be used for most screening situations. Other approaches that could be used for missing items include imputing the missing item with: the mean of the non-missing items of the entire scale for a subject; the mean of the non-missing items of the subscale from which the item is missing, for a subject; the mean of the item over all subjects; and multiple imputation [13].

The question of how to handle missing items for outcomes research has not received as much attention as missing forms, which has a rich history of statistical investigation, and poses different challenges. However, there have been some investigations into missing items for outcomes research. Fayers and colleagues [14] discuss missing items in a quality of life context and give guidelines about imputation, for example, showing when a simple item mean imputation may cause bias. Fairclough and Cella [7] performed an in-depth investigation of various approaches for handling missing items in the FACT-G, resulting in the current recommendations for use of the half-rule.

In order to make valid inferences using the HADS, a principled, evidence-based method of handling missing items is needed. The objective of this study was to investigate seven approaches to handling item non-response, using a large sample of Australian cancer patients and survivors to base simulations on, while assessing sensitivity to overall sample size, proportion of subjects with missing items, the proportion of missing items per subject, and the missingness mechanism.

## Methods

We carried out a simulation study based on real data (described below). One thousand datasets were simulated for each parameter combination: three sample sizes, three missingness mechanisms, three subject-level probabilities for having a missing item, two item-level probabilities for missingness. Description of these parameters follows.

### Data sources

The data originated from two large, related, Australian studies investigating patient reported outcomes, including anxiety and depression, amongst Arabic, Chinese and Greek immigrants as compared to Anglo-Australians cancer survivors and patients. These studies have been described previously [15, 16]. Briefly, the first study recruited survivors from registries (N = 596, response rate = 26 %); the second involved patients, and was hospital based (N = 845, response rate = 61 %). There were 593 Anglo-Australians, 202 Arabic participants, 389 Chinese participants and 257 Greeks participants. Participants had a mix of cancer diagnoses including breast (20 %), colorectal (17 %) and prostate (14 %). Males made up 46 % of the sample. Age ranged from 19 to 87 years with a mean of 63 and standard deviation of 11.8. Immigrants had the choice of completing the form in either English or their native language. Out of the 1441 HADS questionnaires, 1385 (96 %) were complete. Along with the HADS, quality of life was assessed using the Functional Assessment of Cancer Therapy-General (FACT-G), a 27-item questionnaire covering aspects of physical, social, family, emotional, and functional well-being [17].

### Sample size

Beginning with the complete data set (n = 1385), a random sample of subjects was selected, with replacement. We chose starting sample sizes based on detecting standardized effects, d, considered to be large (d = 0.8, n = 52), medium (d = 0.5, n = 128), or small (d = 0.2, n = 788), according to Cohen’s criteria [18], and assuming a 2-sided t test with 80 % power and type I error rate of 0.05.

### Missingness

**Fig. 1:**
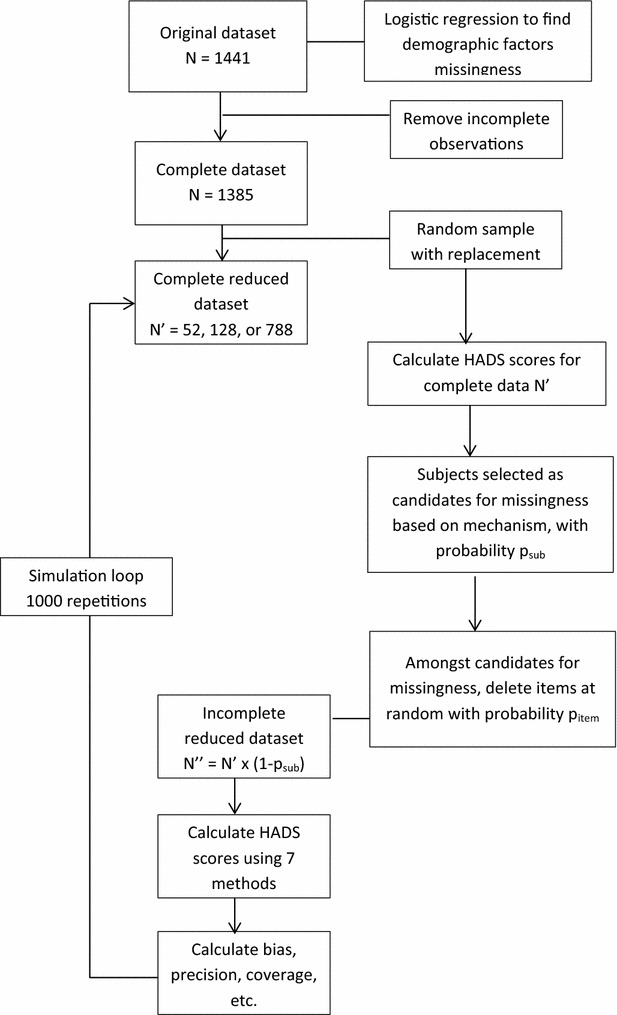
Simulation study flow

To create missingness, items were deleted from the complete data in three ways (i.e., there were three missingness mechanisms): (1) completely random; (2) based on demographic information or (3) based on the subscale’s value (higher values were more likely to be deleted). To mimic the real situation where missing items are clustered by subject, each of the methods used a procedure, based on the missingness mechanism, to select p_sub_ = 10, 20 or 50 % of the subjects to be candidates for item deletion, as detailed below. The probability of missing items within these candidates was then set at p_item_ = 20 or 50 % and item deletion followed by drawing random uniform numbers for each item (range 0–1). If the probability of missing items was set at 20 %, for example, then all items with a random number less than 0.2 would be deleted. The procedure of selecting candidates for missingness (with probability p_sub_) and then randomly selecting items for deletion (with probability = p_item_) resulted in overall missing item rates of 2, 4, 5, 10 and 25 %. These values were chosen to provide a range of missing rates: smaller values that mimicked our data as well as higher values that would discriminate between the methods. The steps of the simulation are shown in Fig. 1.

Random missingness was induced by drawing a random number from the uniform distribution (range 0–1) for each subject. Item deletion within these subjects was then performed by randomly selecting items for deletion with probabilities p_item_ = 20 or 50 %, as described above.

Subscale dependent missingness was carried out by choosing candidates for missingness based on higher subscale scores, so that subjects with higher anxiety, for example, were more likely to have missing items. The highest 10 % (for example) of anxious subjects were candidates for item deletion, which was performed as described above.

Demographic missingness was achieved by increasing the likelihood of deletion based on older age, being on treatment, being male, or being an immigrant. Specifically, each subject’s probability of missing any item (being a missingness candidate) was calculated from a logistic model using the above demographic variables. Subjects with the highest probabilities (e.g., if p_sub_ = 10 %, we used the top 10 %) were then candidates for missing items and item deletion was carried out as in the previous method. The demographic variables were chosen based on predictors of missingness in the original dataset (n = 1441). These variables are specific to our dataset; other datasets are likely to have different predictors of missingness.

### Imputation and scoring

For each dataset there were six ways of imputing missing items: (1) subject’s mean; (2) subject’s subscale mean; (3) subject’s subscale mean if at least half of items were answered (the so-called half-rule); (4) item mean (across all subjects); (5) multiple imputation (MI); and (6) MI if at least half of items are answered. We also scored using a “complete case” approach, where subjects with any missing items were excluded. We used multiple imputation with chained equations (also known as fully conditional specification) for methods 5 and 6, which sequentially imputes missing values using regression [19, 20]. All 14 items were used in the imputation algorithm and imputed items outside the range were truncated (e.g. set to 0 or 3). We created ten complete data sets using SAS Proc MI and averaged the items across the sets to make one complete set from which the anxiety and depression scores are created (see below). This is equivalent to creating ten anxiety and depression scores and combining them using Rubin’s rules to get the point estimate (which is just the average of the estimates) [21].^1^ Each of the methods were chosen based on their current use by researchers or their ease of use.

The standard scoring algorithm was used: anxiety score = sum of items 1*, 3*, 5*, 7, 9, 11*, 13*; and depression = sum of items 2, 4, 6*, 8*, 10*, 12, 14 where starred items are reverse scored. Both subscales have a possible range of 0–21, with higher scores indicating higher anxiety and/or depression. The anxiety and depression scores from the complete (but reduced, n = 52, 128, 788) dataset were calculated in order to assess the performance of the other methods. Thus for each dataset and its subset with the missing items, eight anxiety and depression scores were calculated.

### Statistical methods

We assessed each method by considering performance with respect to both individual and population scores. For individual scores, bias was assessed by computing the average difference of the individual’s imputed and observed (complete) subscale or total score and imprecision was assessed by averaging the squared differences.

For the population, bias was measured as the difference between the mean imputed score (or case-wise deleted score) and the mean complete score in the sample. Imprecision was calculated as the squared difference. A difference of 10 % of a scale is sometimes considered to be the minimum important difference (MID) [22], so we used 10 % of the subscale (2.1 points) to indicate an important level of bias. At the suggestion of a reviewer, correlation with quality of life was also estimated.

## Results

Descriptive statistics for the original sample are given in Table 1. The mean anxiety score was 5.66 with a standard deviation of 4.20; the mean depression score was 5.07 with a standard deviation of 4.11. Most participants were in the normal range (0–7) for both anxiety (73.6 %) and depression (76.1 %); 17.2 and 16.6 % were in the mild range (8–10); 7.0 and 5.9 % were in the moderate range (11–14); and 2.1 and 1.5 % were in the severe range (15–21) for anxiety and depression respectively. Cronbach’s alpha was 0.87, 0.83 and 0.90 for anxiety, depression and distress respectively. Missing HADS item rates ranged from 1.7 to 2.1 %.

**Table 1:** Descriptive statistics for the Hospital Anxiety and Depression Score (HADS), and correlation with quality of life (QoL) for 1444 Australian cancer patients and survivors

Item number	Item content	Number missing	% missing	Mean^a^	Standard deviation	Correlation with total^b^	Correlation with QoL^c^
Anxiety subscale (Cronbach’s alpha = 0.87)				5.66	4.20		−0.687
1	I feel tense or wound up	26	1.8	0.87	0.77	0.69	
3	I get a sort of frightened feeling, as if something awful is about to happen	26	1.8	0.86	0.83	0.70	
5	Worrying thoughts go through my head	25	1.7	1.00		0.68	
7	I can sit at ease and feel relaxed	24	1.7	0.92	0.77	0.62	
9	I get a frightened feeling like butterflies in the stomach	31	2.1	0.56	0.73	0.60	
11	I feel restless as if I have to be on the move	28	1.9	0.87	0.84	0.50	
13	I get sudden feelings of panic	28	1.9	0.44	0.72	0.73	
Depression subscale (Cronbach’s alpha = 0.84)				5.07	4.11		−0.767
2	I still enjoy things I used to enjoy	24	1.7	0.74	0.83	0.58	
4	I can laugh and see the funny side of things	28	1.9	0.51	0.76	0.64	
6	I feel cheerful	24	1.7	0.65	0.75	0.68	
8	I feel as if I am slowed down	28	1.9	1.36	0.92	0.50	
10	I have lost interest in my appearance	29	2.0	0.75	0.98	0.51	
12	I look forward with enjoyment to things	28	1.9	0.66	0.84	0.71	
14	I can enjoy a good book or radio or TV program	28	1.9	0.44	0.72	0.49	
Distress total score (Cronbach’s alpha = 0.90)				10.74	7.60		−0.796

Higher values indicate higher anxiety or depression

^a^Possible range of each item is 0–3; range for subscales is 0–21; range for distress is 0–42

^b^Correlation of item with total of remaining items in subscale

^c^QoL is measured by the FACT-G

### Simulation results: individual scores

Results for the depression subscale are shown in Table 2, for n = 52, since results did not vary by sample size. Full results including anxiety, distress, each of the missing item rates and each of the sample sizes are given in Additional file 1: Appendix S1.

**Table 2:** Mean bias and imprecision of *individual* scores for depression, n = 52 for random, demographic and subscale dependent missingness mechanisms

Method	Bias			Imprecision			n method^a^
	Random	Demog	Subscale	Random	Demog	Subscale	
25 % missing item rate, p_sub_ = 0.5 p_item_ = 0.5							
Subject mean	0.083	0.002	−0.060	1.638	1.707	2.177	52
Subscale mean	0.004	−0.007	−0.010	2.316	2.533	3.483	52
Subscale ½ mean	0.006	−0.002	−0.003	0.617	0.637	0.886	40
Item mean	0.001	−0.360	−1.110	2.755	3.453	4.745	52
Multiple imputation	0.016	−0.087	−0.324	1.727	2.043	2.623	52
Multiple imputation ½	0.010	−0.027	−0.119	0.635	0.738	0.965	40
10 % missing item rate, p_sub_ = 0.5 p_item_ = 0.2							
Subject mean	0.028	0.002	−0.025	0.459	0.474	0.632	52
Subscale mean	−0.003	0.001	−0.002	0.526	0.543	0.767	52
Subscale ½ mean	−0.002	0.001	−0.000	0.437	0.453	0.631	47
Item mean	−0.002	−0.123	−0.372	0.747	0.897	1.052	52
Multiple imputation	0.005	−0.012	−0.075	0.532	0.612	0.773	52
Multiple imputation ½	0.001	−0.007	−0.063	0.472	0.545	0.672	47
10 % missing item rate, p_sub_ = 0.2 p_item_ = 0.5							
Subject mean	0.034	−0.003	−0.082	0.674	0.685	0.991	52
Subscale mean	−0.001	−0.004	0.001	0.986	1.025	1.468	52
Subscale ½ mean	0.004	−0.002	−0.001	0.212	0.212	0.313	47
Item mean	−0.002	−0.132	−0.702	1.102	1.310	3.435	52
Multiple imputation	0.005	−0.027	−0.234	0.662	0.751	1.276	52
Multiple imputation ½	0.002	−0.007	−0.074	0.195	0.222	0.363	47
2 % missing item rate, p_sub_ = 0.1 p_item_ = 0.2							
Subject mean	0.006	−0.000	−0.023	0.091	0.098	0.151	52
Subscale mean	−0.000	−0.000	0.001	0.106	0.110	0.158	52
Subscale ½ mean	0.000	0.000	0.001	0.083	0.086	0.128	51
Item mean	0.000	−0.029	−0.164	0.141	0.177	0.541	52
Multiple imputation	0.001	−0.004	−0.038	0.101	0.112	0.192	52
Multiple imputation ½	0.000	−0.002	−0.033	0.086	0.193	0.163	51

1000 simulated datasets

^a^Sample size after imputation

The methods were most sensitive within the subscale missingness mechanism, with higher values of bias and imprecision than the mechanisms of demographic and random, which had similar values. The method that consistently yielded the lowest imprecision and bias for individual scores was the subscale half mean. The next best method for bias was the subscale mean, and the MI ½ for imprecision. The worst method was the item mean, followed by MI and the subject mean, which were similar. These results were consistent regardless of outcome (depression, anxiety, distress), overall sample size, proportion of subjects with missing items, the proportion of missing items per subject, and the missingness mechanism.

### Simulation results: population means

Results for population means, at 10 and 25 % missing item rate, are given in Table 3. Results were not dependent on sample size, so only n = 52 is shown. Similar to the individual scores, methods were the most sensitive within the subscale missingness mechanism, with higher values of bias and imprecision than the random and demographic missingness. The correlation with QoL was highly effected when no imputation was used for subscale dependent missingness. For example, correlation was estimated at −0.427 for n = 52 and p_item_ = 0.5, when the correlation for the entire sample was −0.767. The other estimates for this proportion of missing data ranged from −0.701 to −0.769. Although estimated correlations were not as disparate for smaller rates of missing data, the magnitude of the correlation was consistently underestimated.

**Table 3:** Bias and imprecision of *population* means for the HADS depression subscale, and correlation with quality of life

Method	Bias			Imprecision			Correlation with QoL^a^		
	Random	Demog	Subscale	Random	Demog	Subscale	Random	Demog	Subscale
25 % missing item, rate = p_sub_ = 0.5 p_item_ = 0.5									
Subject mean	0.083	0.002	−0.061	0.037	0.033	0.047	−0.754	−0.759	−0.752
Subscale mean	0.004	−0.011	−0.024	0.046	0.051	0.071	−0.722	−0.715	−0.701
Subscale ½ mean	0.000	−0.357	−1.102	0.141	0.258	1.377	−0.756	−0.757	−0.729
Item mean	0.001	−0.360	−1.110	0.062	0.189	1.277	−0.756	−0.721	−0.717
Multiple imputation	0.016	−0.086	−0.324	0.038	0.053	0.175	−0.711	−0.744	−0.729
Multiple imputation ½	0.005	−0.382	−1.218	0.136	0.274	1.638	−0.742	−0.769	−0.732
Complete case	−0.016	−1.026	−3.168	0.348	1.347	10.162	−0.769	−0.769	−0.427
10 % missing item rate, p_sub_ = 0.5 p_item_ = 0.2									
Subject mean	0.028	0.002	−0.025	0.010	0.009	0.013	−0.768	−0.769	−0.768
Subscale mean	−0.003	0.001	−0.002	0.010	0.010	0.015	−0.760	−0.768	−0.754
Subscale ½ mean	−0.000	−0.025	−0.067	0.016	0.019	0.026	−0.769	−0.768	−0.743
Item mean	−0.002	−0.123	−0.372	0.014	0.030	0.154	−0.758	−0.755	−0.743
Multiple imputation	0.005	−0.012	−0.075	0.011	0.012	0.023	−0.766	−0.765	−0.765
Multiple imputation ½	0.003	−0.033	−0.130	0.016	0.021	0.040	−0.770	−0.769	−0.746
Complete case	−0.000	−0.695	−2.122	0.201	0.699	4.720	−0.770	−0.770	−0.716
10 % missing item rate, p_sub_ = 0.2 p_item_ = 0.5									
Subject mean	0.034	0.003	−0.082	0.014	0.014	0.025	−0.764	−0.767	−0.765
Subscale mean	0.000	−0.006	−0.010	0.018	0.020	0.028	−0.750	−0.747	−0.740
Subscale ½ mean	0.002	−0.134	−0.701	0.041	0.064	0.575	−0.764	−0.766	−0.730
Item mean	−0.002	−0.132	−0.702	0.022	0.041	0.514	−0.745	−0.746	−0.731
Multiple imputation	−0.005	−0.027	−0.234	0.013	0.015	0.080	−0.761	−0.761	−0.758
Multiple imputation ½	−0.000	−0.139	−0.774	0.040	0.064	0.672	−0.766	−0.769	−0.734
Complete case	−0.003	−0.285	−1.540	0.077	0.161	2.409	−0.768	−0.772	−0.666
2 % missing item rate, p_sub_ = 0.1 p_item_ = 0.2									
Subject mean	0.006	−0.000	−0.023	0.002	0.002	0.003	−0.769	−0.771	−0.771
Subscale mean	−0.000	−0.000	0.001	0.002	0.002	0.003	−0.767	−0.767	−0.767
Subscale ½ mean	0.000	−0.009	−0.034	0.003	0.004	0.010	−0.768	−0.768	−0.766
Item mean	0.000	−0.029	−0.166	0.003	0.004	0.033	−0.766	−0.766	−0.767
Multiple imputation	0.001	−0.004	−0.038	0.002	0.002	0.005	−0.768	−0.769	−0.769
Multiple imputation ½	0.001	−0.01	−0.068	0.003	0.004	0.014	−0.769	−0.769	−0.768
Complete case	−0.000	−0.119	−0.679	0.026	0.043	0.500	−0.770	−0.770	−0.729

N = 52 for random, demographic dependent and subscale dependent missingness mechanisms. 1000 simulated datasets

^a^Correlation = −0.767 for original data (n = 1444)

The worst methods for bias and imprecision were those that resulted in a reduced number of individuals with scores, and the item mean. The worst performing was the complete case. This was largely consistent regardless of outcome (depression, anxiety, distress), overall sample size, proportion of subjects with missing items, the proportion of missing items per subject, and the missingness mechanism. The best method for bias and imprecision was the subject mean, followed by the subscale mean and MI. The magnitude of the bias and imprecision was independent of sample size. Bias ranged from 0 (subject mean) to −3.2 (complete case, subscale missingness, 25 % missing rate). The largest bias amongst the imputation methods was about −1.1 to −1.2, for both the half methods, which is slightly less than the pre-specified 2.1 point importance criteria.

Bias and imprecision were not affected by sample size, but they did vary slightly by missingness rates, and by p_sub_ and p_item_. However, comparing the two cases where the missing item rate was 10 %, we see that the worst methods, overall, were still the complete case, item mean, and the half methods. At 2 % missing item rate, the missing item rate of the source data, bias and imprecision is very small.

## Discussion

We performed an extensive simulation study to investigate the best of seven approaches for handling missing items in the HADS. We varied the missingness mechanism, the overall sample size, proportion of subjects with missing items, and the proportion of missing items per subject. We assessed the methods based on both population and individual values. All imputation methods were superior to omitting subjects with missing data (complete case analysis). The best performing imputation methods depended on whether inference was targeted at the individual or at the population. For individuals, the top performing method was the subscale half mean. This method, however, performed poorly according to population measures, with higher bias and imprecision when the proportions of missing data were high. The best method for population inference was the subject mean. However, these issues mostly disappeared as the proportions approached the levels observed in the source data (~2 %). This is consistent with the lack of bias at the individual level particularly for the method that used the subscale mean.

To further investigate the effect of high numbers of missing items within an individual, we conducted another small simulation study to compare the subscale mean and the subscale half mean methods for population measures. We let p_item_ range from 0.5 to 0.929, which corresponds to 7–13 missing items out of the 14. We used p_sub_ = 0.1 and 0.5 (=probability a subject has a missing item) and 1000 simulated datasets of n = 52 with subscale dependent missingness mechanism. We found that when p_sub_ = 0.1, both methods worked well for bias, even with high numbers of missing items. When p_sub_ = 0.5 the subscale mean performed well, in terms of bias and imprecision for up to 12 missing items. The half mean method broke down much sooner. For example, with nine missing items, the bias for the subscale mean was −0.10, as compared to −2.19 for the half method. This indicates that very few complete items may be needed, if inference is population based. Full results can be found in Additional file 2: Appendix S2.

The relatively strong performance of the subscale half mean relative to MI for individuals is likely to have occurred because our study assumed that particular items in the HADS were not more likely to be missing than others, an assumption borne out by examination of missing item rates in the original dataset. If missingness had been particularly high for the items with low (or high) overall means, it may not have performed as well [14]. This uniform missingness is not always the case for all questionnaires. For example, Bell et al. [23] showed that items concerning sexuality were more likely to be missing, and missing informatively, in the FACT-G and the Supportive Care Needs Survey [24]. For questionnaires with varying levels of difficulty, and therefore potential for differential missingness, item response theory may be more appropriate [25] though implementation will be a challenge in settings with limited computational resources.

A strength of this study is the large sample size amongst a diverse population, with both cancer patients and survivors, and varying ethnicity. The standard deviations of 4.11 for anxiety, 4.19 for depression and 7.60 for distress are similar to other psychosocial research studies [26], indicating that the study is likely to be generalizable. Another strength is the investigation into performance at both the individual and population level. A limitation is that our study was based on individuals affected by cancer and it is possible that results could vary for different conditions. In particular, if these individuals were more distressed than other populations there would be more right skewness in this sample, which would make the item mean imputation more biased towards higher distress. This would not affect imputation methods based on a subject’s own mean. In practice, the true missing mechanism can be difficult or impossible to determine. Furthermore, missingness is unlikely to be due to a single mechanism. The simulations we have conducted show the extreme cases: random missingness, where the effect of missingness is very small, to subscale dependent missingness, where the effect is larger. In a study, where there are multiple mechanisms, bias and imprecision is likely to fall somewhere in between the two extremes we have shown.

Some researchers use the HADS to classify patients into “depressed” or “anxious” based on a cutoff of eight points [4]. It is well known that dichotomizing continuous variables can lead to problems including misclassification bias [27], and lower power. Given the consistent underestimation of depression in this study, the likelihood of misclassifying depressed (or anxious) individuals as not depressed (or anxious) is increased, although only very slightly for small rates of missingness, and primarily for the complete case approach.

Our objective was to investigate handling missing items in a particular questionnaire, the HADS, so that the subscales or total score can be used for either screening or analyses, such as regression models. If other variables or the entire HADS questionnaire are missing, one may consider using multiple imputation, at least as a sensitivity analysis [5, 28].

## Conclusions

Based on these simulations, we strongly recommend the ‘half rule’ using individual subscale means when using the HADS scores at the individual level (e.g. screening). For investigations relying on summary statistics (e.g. sample means), either individual subject, subscale means or MI would be preferable, although we prefer the subject or subscale means due to the comparative simplicity of use. The issue of whether to impose the ‘half rule’ may be academic for studies such as those we used as our source data, as the proportions of subjects who would have more than half the items missing are often quite small. When missing item rates increased, however, important levels of bias occurred, both in the mean of the HADS and the correlation with QoL, underscoring the importance of avoiding missing data.

## Additional files

**Additional file 1: Appendix S1.** Individual results.

**Additional file 2: Appendix S2.** Simulation increasing number of missing items.

## Abbreviations

FACT-G: Functional Assessment of Cancer Therapy General
HADS: Hospital Anxiety and Depression Scale
MI: multiple imputation
SD: standard deviation
SF36: Short Form 36
QoL: quality of life

## Footnotes

1 MI generally proceeds as follows: (1) Create M complete datasets. (2) Analyze each of the datasets and get an estimate. (3) Combine M estimates using Rubin’s rules. The point estimate is the average of the M estimates. Since we are not using the variance estimates, it is equivalent to average the items across M multiply imputed datasets and then create one score. Let ${\text{m}} = 1, \ldots ,{\text{M}}$ imputations, ${\text{i}} = 1, \ldots {\text{k}}$ items X and *H*_*m*_ = the HADS score for the mth imputed dataset = $\sum\nolimits_{i = 1}^{k} {X_{mi} }$. Then the combined estimate of the HADS score is $H = \tfrac{1}{M}\sum\nolimits_{m}^{M} {H_{m} } = \tfrac{1}{M}\sum\nolimits_{m = 1}^{M} {\sum\nolimits_{i = 1}^{k} {X_{mi} } = } \sum\nolimits_{i = 1}^{k} {\tfrac{1}{M}\sum\nolimits_{m = 1}^{M} {X_{mi} } }$. We are not using the variance estimates in this simulation because we are not performing analyses.
