# Supplementary material for: Handling missing items in the Hospital Anxiety and Depression Scale (HADS): a simulation study
Source: BMC Res Notes. 2016 Oct 22;9:479. doi: 10.1186/s13104-016-2284-z (PMC5075158; doi:10.1186/s13104-016-2284-z)
Supplement: Supplementary file 1 — Additional file 1: Appendix S1. Individual results. [file 13104_2016_2284_MOESM1_ESM.rtf]

	Bias	Imprecision	
	Random	Demog	Subscale	Random	Demog	Subscale	
N		-0.006	-0.000	0.024	0.073	0.070	0.120	
52	Subject mean							
	Subscale mean	-0.001	0.000	-0.001	0.077	0.071	0.111	
	Subscale ½ mean	-0.000	0.000	-0.000	0.068	0.060	0.095	
	Item mean	0.002	-0.015	-0.105	0.135	0.153	0.345	
	Multiple imputation	0.001	0.005	0.005	0.083	0.087	0.138	
	Multiple imputation ½	0.001	0.004	0.006	0.075	0.076	0.119	
128	Subject mean	-0.007	0.001	0.025	0.071	0.069	0.118	
	Subscale mean	-0.000	0.001	0.001	0.073	0.068	0.112	
	Subscale ½ mean	0.000	0.001	0.000	0.063	0.059	0.094	
	Item mean	-0.000	-0.015	-0.106	0.139	0.156	0.346	
	Multiple imputation	0.001	0.003	0.003	0.068	0.068	0.104	
	Multiple imputation ½	0.000	0.003	0.003	0.060	0.061	0.091	
788	Subject mean	-0.009	0.001	0.032	0.098	0.097	0.153	
	Subscale mean	-0.001	0.000	0.001	0.100	0.094	0.144	
	Subscale ½ mean	-0.000	0.000	0.001	0.085	0.080	0.122	
	Item mean	-0.001	-0.021	-0.126	0.184	0.209	0.406	
	Multiple imputation	-0.000	0.003	0.005	0.084	0.084	0.118	
	Multiple imputation ½	0.000	0.003	0.004	0.074	0.072	0.104	

	Bias	Imprecision	
	Random	Demog	Subscale	Random	Demog	Subscale	
N		-0.015	-0.002	0.058	0.284	0.287	0.468	
52	Subject mean							
	Subscale mean	0.001	-0.000	-0.002	0.351	0.313	0.488	
	Subscale ½ mean	0.000	-0.001	0.002	0.070	0.067	0.108	
	Item mean	0.001	-0.043	-0.276	0.549	0.620	1.531	
	Multiple imputation	0.003	0.005	-0.002	0.286	0.314	0.466	
	Multiple imputation ½	0.001	0.001	0.000	0.080	0.081	0.121	
128	Subject mean	-0.014	0.002	0.060	0.286	0.298	0.465	
	Subscale mean	0.002	-0.001	-0.002	0.338	0.299	0.493	
	Subscale ½ mean	0.001	-0.000	-0.001	0.073	0.066	0.105	
	Item mean	-0.003	-0.042	-0.279	0.543	0.629	1.546	
	Multiple imputation	0.002	0.005	-0.004	0.252	0.259	0.383	
	Multiple imputation ½	0.002	0.002	-0.001	0.068	0.067	0.098	
788	Subject mean	-0.022	0.001	0.080	0.388	0.388	0.612	
	Subscale mean	-0.001	-0.001	0.001	0.454	0.422	0.652	
	Subscale ½ mean	-0.000	-0.000	0.000	0.098	0.092	0.143	
	Item mean	-0.002	-0.055	-0.329	0.721	0.830	1.764	
	Multiple imputation	-0.001	0.004	0.003	0.318	0.320	0.455	
	Multiple imputation ½	0.000	0.002	0.001	0.084	0.082	0.118	

	Bias	Imprecision	
	Random	Demog	Subscale	Random	Demog	Subscale	
N		-0.013	-0.001	0.032	0.147	0.143	0.211	
52	Subject mean							
	Subscale mean	0.001	0.001	-0.002	0.150	0.143	0.203	
	Subscale ½ mean	0.001	0.001	-0.000	0.128	0.122	0.175	
	Item mean	0.002	-0.025	-0.174	0.277	0.309	0.537	
	Multiple imputation	0.003	0.008	0.005	0.176	0.176	0.252	
	Multiple imputation ½	0.003	0.006	0.003	0.156	0.158	0.221	
128	Subject mean	-0.012	-0.000	0.036	0.153	0.150	0.217	
	Subscale mean	0.002	0.002	0.001	0.156	0.150	0.214	
	Subscale ½ mean	0.002	0.002	0.001	0.132	0.127	0.180	
	Item mean	-0.001	-0.024	-0.177	0.293	0.314	0.544	
	Multiple imputation	0.003	0.005	0.005	0.145	0.146	0.198	
	Multiple imputation ½	0.002	0.005	0.005	0.127	0.128	0.172	
788	Subject mean	-0.017	-0.000	0.037	0.196	0.199	0.267	
	Subscale mean	0.000	0.000	0.001	0.202	0.203	0.265	
	Subscale ½ mean	-0.000	0.000	0.001	0.171	0.172	0.225	
	Item mean	0.002	-0.028	-0.208	0.373	0.403	0.641	
	Multiple imputation	0.002	0.007	0.003	0.170	0.178	0.217	
	Multiple imputation ½	0.001	0.006	0.002	0.148	0.155	0.190	

	Bias	Imprecision	
	Random	Demog	Subscale	Random	Demog	Subscale	
N		-0.030	-0.004	0.078	0.588	0.578	0.834	
52	Subject mean							
	Subscale mean	0.004	0.003	-0.008	0.700	0.634	0.912	
	Subscale ½ mean	0.001	-0.000	0.000	0.154	0.148	0.209	
	Item mean	-0.001	-0.070	-0.467	1.116	1.233	2.404	
	Multiple imputation	0.010	0.016	-0.007	0.614	0.633	0.892	
	Multiple imputation ½	0.004	0.004	0.003	0.171	0.184	0.248	
128	Subject mean	-0.035	-0.003	0.087	0.611	0.618	0.842	
	Subscale mean	-0.002	-0.000	-0.001	0.699	0.654	0.946	
	Subscale ½ mean	0.001	-0.001	-0.002	0.162	0.149	0.216	
	Item mean	0.002	-0.066	-0.475	1.153	1.245	2.410	
	Multiple imputation	0.002	0.012	-0.001	0.534	0.543	0.732	
	Multiple imputation ½	0.002	0.002	-0.001	0.151	0.151	0.199	
788	Subject mean	-0.040	-0.001	0.090	0.768	0.783	1.056	
	Subscale mean	0.001	-0.001	0.002	0.912	0.907	1.193	
	Subscale ½ mean	0.001	-0.001	0.001	0.212	0.209	0.278	
	Item mean	0.001	-0.077	-0.568	1.450	1.585	2.834	
	Multiple imputation	0.003	0.016	-0.004	0.637	0.656	0.832	
	Multiple imputation ½	0.002	0.005	0.000	0.181	0.185	0.231	

	Bias	Imprecision	
	Random	Demog	Subscale	Random	Demog	Subscale	
N		-0.035	-0.005	0.020	0.361	0.373	0.446	
52	Subject mean							
	Subscale mean	-0.004	0.000	-0.001	0.367	0.376	0.459	
	Subscale ½ mean	-0.002	0.001	-0.001	0.317	0.323	0.392	
	Item mean	-0.001	-0.056	-0.256	0.709	0.764	0.904	
	Multiple imputation	0.002	0.018	0.001	0.446	0.481	0.584	
	Multiple imputation ½	0.002	0.017	0.002	0.393	0.426	0.513	
128	Subject mean	-0.034	0.003	0.022	0.388	0.401	0.468	
	Subscale mean	0.001	0.004	0.002	0.397	0.406	0.485	
	Subscale ½ mean	0.001	0.004	0.001	0.338	0.348	0.413	
	Item mean	0.001	-0.059	-0.253	0.743	0.797	0.916	
	Multiple imputation	0.003	0.020	0.000	0.377	0.402	0.457	
	Multiple imputation ½	0.003	0.015	0.000	0.332	0.355	0.404	
788	Subject mean	-0.040	-0.010	0.004	0.495	0.503	0.565	
	Subscale mean	0.002	0.001	0.002	0.510	0.521	0.586	
	Subscale ½ mean	0.001	0.001	0.002	0.435	0.445	0.503	
	Item mean	0.000	-0.043	-0.225	0.938	0.990	1.043	
	Multiple imputation	0.004	0.016	-0.002	0.429	0.445	0.486	
	Multiple imputation ½	0.003	0.015	-0.002	0.378	0.393	0.429	

	Bias	Imprecision	
	Random	Demog	Subscale	Random	Demog	Subscale	
N		-0.085	-0.007	0.050	1.417	1.471	1.782	
52	Subject mean							
	Subscale mean	-0.001	0.004	-0.004	1.675	1.727	2.027	
	Subscale ½ mean	0.000	0.002	-0.002	0.448	0.462	0.563	
	Item mean	-0.009	-0.167	-0.771	2.803	3.038	4.033	
	Multiple imputation	0.010	0.051	-0.024	1.568	1.726	2.109	
	Multiple imputation ½	0.003	0.023	-0.009	0.549	0.595	0.722	
128	Subject mean	-0.076	0.001	0.054	1.511	1.591	1.811	
	Subscale mean	0.010	0.005	0.003	1.788	1.829	2.147	
	Subscale ½ mean	0.005	0.001	-0.002	0.478	0.493	0.589	
	Item mean	0.005	-0.181	-0.765	2.869	3.141	4.026	
	Multiple imputation	0.017	0.053	-0.010	1.374	1.487	1.717	
	Multiple imputation ½	0.008	0.021	-0.008	0.465	0.503	0.591	
788	Subject mean	-0.105	-0.026	0.009	1.922	1.946	2.232	
	Subscale mean	-0.002	0.000	0.002	2.287	2.323	2.669	
	Subscale ½ mean	-0.001	0.000	0.001	0.693	0.696	0.793	
	Item mean	-0.003	-0.140	-0.728	3.638	3.871	4.416	
	Multiple imputation	0.003	0.050	-0.019	1.595	1.651	1.850	
	Multiple imputation ½	0.002	0.023	-0.009	0.594	0.612	0.680	

	Bias	Imprecision	
	Random	Demog	Subscale	Random	Demog	Subscale	
N		0.006	-0.000	-0.023	0.091	0.098	0.151	
52	Subject mean							
	Subscale mean	-0.000	-0.000	0.001	0.106	0.110	0.158	
	Subscale ½ mean	0.000	0.000	0.001	0.083	0.086	0.128	
	Item mean	0.000	-0.029	-0.164	0.141	0.177	0.541	
	Multiple imputation	0.001	-0.004	-0.038	0.101	0.115	0.197	
	Multiple imputation ½	0.000	-0.002	-0.033	0.086	0.093	0.163	
128	Subject mean	0.006	-0.000	-0.024	0.095	0.100	0.148	
	Subscale mean	-0.001	0.000	-0.000	0.110	0.114	0.160	
	Subscale ½ mean	0.000	0.001	0.000	0.089	0.094	0.130	
	Item mean	-0.001	-0.030	-0.167	0.142	0.172	0.547	
	Multiple imputation	0.000	-0.003	-0.038	0.085	0.096	0.158	
	Multiple imputation ½	-0.000	-0.003	-0.032	0.074	0.085	0.129	
788	Subject mean	0.009	-0.000	-0.032	0.124	0.136	0.197	
	Subscale mean	-0.000	-0.000	-0.001	0.142	0.154	0.215	
	Subscale ½ mean	-0.000	-0.000	-0.001	0.118	0.128	0.178	
	Item mean	0.000	-0.038	-0.204	0.190	0.218	0.630	
	Multiple imputation	0.001	-0.006	-0.046	0.101	0.119	0.183	
	Multiple imputation ½	0.001	-0.005	-0.040	0.088	0.104	0.155	

	Bias	Imprecision	
	Random	Demog	Subscale	Random	Demog	Subscale	
N		0.015	0.001	-0.059	0.321	0.348	0.542	
52	Subject mean							
	Subscale mean	-0.004	-0.002	-0.002	0.477	0.505	0.736	
	Subscale ½ mean	0.002	0.000	0.002	0.100	0.103	0.148	
	Item mean	-0.000	-0.074	-0.423	0.547	0.664	2.351	
	Multiple imputation	0.000	-0.016	-0.142	0.319	0.380	0.729	
	Multiple imputation ½	0.001	-0.003	-0.041	0.094	0.102	0.184	
128	Subject mean	0.017	-0.000	-0.061	0.332	0.359	0.556	
	Subscale mean	0.001	0.001	-0.001	0.478	0.524	0.711	
	Subscale ½ mean	0.001	-0.000	0.002	0.101	0.114	0.153	
	Item mean	-0.005	-0.079	-0.429	0.537	0.647	2.385	
	Multiple imputation	0.001	-0.017	-0.137	0.284	0.333	0.641	
	Multiple imputation ½	-0.000	-0.004	-0.038	0.080	0.099	0.153	
788	Subject mean	0.020	-0.003	-0.080	0.436	0.479	0.718	
	Subscale mean	-0.001	-0.002	-0.001	0.623	0.682	0.970	
	Subscale ½ mean	-0.000	-0.000	-0.000	0.136	0.149	0.213	
	Item mean	-0.001	-0.098	-0.530	0.707	0.815	2.795	
	Multiple imputation	0.000	-0.024	-0.170	0.344	0.406	0.764	
	Multiple imputation ½	0.000	-0.006	-0.049	0.097	0.116	0.189	

	Bias	Imprecision	
	Random	Demog	Subscale	Random	Demog	Subscale	
N		0.010	0.002	-0.034	0.185	0.188	0.282	
52	Subject mean							
	Subscale mean	-0.004	0.000	-0.001	0.210	0.215	0.315	
	Subscale ½ mean	-0.002	0.001	0.001	0.171	0.175	0.258	
	Item mean	0.001	-0.050	-0.266	0.295	0.342	0.776	
	Multiple imputation	-0.000	-0.003	-0.061	0.208	0.229	0.354	
	Multiple imputation ½	-0.001	-0.002	-0.052	0.183	0.198	0.298	
128	Subject mean	0.014	0.002	-0.034	0.195	0.204	0.287	
	Subscale mean	-0.001	0.000	0.001	0.219	0.230	0.331	
	Subscale ½ mean	-0.000	0.001	0.002	0.181	0.192	0.274	
	Item mean	-0.001	-0.048	-0.272	0.306	0.345	0.786	
	Multiple imputation	0.001	-0.003	-0.058	0.176	0.200	0.294	
	Multiple imputation ½	0.001	-0.004	-0.049	0.152	0.173	0.249	
788	Subject mean	0.016	-0.000	-0.037	0.247	0.260	0.360	
	Subscale mean	-0.001	-0.001	-0.002	0.281	0.299	0.411	
	Subscale ½ mean	0.000	-0.000	-0.001	0.236	0.248	0.344	
	Item mean	0.002	-0.061	-0.314	0.383	0.452	0.860	
	Multiple imputation	0.001	-0.007	-0.064	0.202	0.238	0.319	
	Multiple imputation ½	0.002	-0.006	-0.057	0.178	0.207	0.278	

	Bias	Imprecision	
	Random	Demog	Subscale	Random	Demog	Subscale	
N		0.034	0.003	-0.082	0.674	0.685	0.991	
52	Subject mean							
	Subscale mean	-0.001	-0.004	0.001	0.986	1.025	1.468	
	Subscale ½ mean	0.004	-0.002	-0.001	0.212	0.212	0.313	
	Item mean	-0.002	-0.132	-0.702	1.102	1.310	3.435	
	Multiple imputation	0.005	-0.027	-0.236	0.662	0.750	1.280	
	Multiple imputation ½	0.002	-0.007	-0.074	0.195	0.222	0.363	
128	Subject mean	0.031	0.004	-0.090	0.696	0.726	1.056	
	Subscale mean	-0.004	-0.001	-0.005	0.982	1.038	1.487	
	Subscale ½ mean	-0.000	-0.001	0.001	0.218	0.236	0.342	
	Item mean	0.002	-0.130	-0.728	1.137	1.319	3.537	
	Multiple imputation	0.002	-0.024	-0.226	0.593	0.679	1.135	
	Multiple imputation ½	0.003	-0.005	-0.068	0.173	0.203	0.315	
788	Subject mean	0.042	-0.002	-0.091	0.884	0.923	1.269	
	Subscale mean	-0.001	-0.004	-0.002	1.265	1.328	1.846	
	Subscale ½ mean	-0.001	-0.001	-0.002	0.296	0.310	0.431	
	Item mean	0.001	-0.165	-0.853	1.429	1.707	3.921	
	Multiple imputation	0.003	-0.035	-0.242	0.699	0.818	1.212	
	Multiple imputation ½	0.001	-0.008	-0.075	0.214	0.249	0.348	

	Bias	Imprecision	
	Random	Demog	Subscale	Random	Demog	Subscale	
N		0.028	0.002	-0.025	0.459	0.474	0.632	
52	Subject mean							
	Subscale mean	-0.003	0.001	-0.002	0.526	0.543	0.767	
	Subscale ½ mean	-0.002	0.001	-0.000	0.437	0.453	0.631	
	Item mean	-0.002	-0.123	-0.372	0.747	0.897	1.052	
	Multiple imputation	0.005	-0.010	-0.074	0.532	0.620	0.775	
	Multiple imputation ½	0.001	-0.007	-0.063	0.472	0.545	0.672	
128	Subject mean	0.034	0.004	-0.022	0.481	0.512	0.659	
	Subscale mean	0.001	0.001	-0.001	0.551	0.578	0.788	
	Subscale ½ mean	0.002	0.000	0.001	0.464	0.495	0.671	
	Item mean	0.002	-0.125	-0.368	0.750	0.910	1.057	
	Multiple imputation	0.006	-0.012	-0.065	0.444	0.518	0.640	
	Multiple imputation ½	0.006	-0.010	-0.056	0.390	0.468	0.571	
788	Subject mean	0.043	0.010	-0.004	0.614	0.638	0.799	
	Subscale mean	0.000	-0.001	-0.002	0.704	0.734	0.961	
	Subscale ½ mean	-0.000	-0.001	-0.001	0.598	0.624	0.816	
	Item mean	0.001	-0.110	-0.323	0.950	1.066	1.113	
	Multiple imputation	0.005	-0.014	-0.058	0.507	0.564	0.674	
	Multiple imputation ½	0.005	-0.012	-0.051	0.451	0.500	0.600	

	Bias	Imprecision	
	Random	Demog	Subscale	Random	Demog	Subscale	
N		0.083	0.002	-0.060	1.638	1.707	2.177	
52	Subject mean							
	Subscale mean	0.004	-0.007	-0.010	2.316	2.533	3.483	
	Subscale ½ mean	0.006	-0.002	-0.003	0.617	0.637	0.886	
	Item mean	0.001	-0.360	-1.110	2.755	3.453	4.745	
	Multiple imputation	0.016	-0.086	-0.318	1.727	2.056	2.595	
	Multiple imputation ½	0.010	-0.027	-0.119	0.635	0.738	0.965	
128	Subject mean	0.085	0.004	-0.055	1.726	1.816	2.292	
	Subscale mean	0.003	-0.004	-0.007	2.472	2.627	3.524	
	Subscale ½ mean	0.000	-0.004	-0.000	0.680	0.725	0.983	
	Item mean	-0.001	-0.389	-1.113	2.850	3.570	4.762	
	Multiple imputation	0.009	-0.074	-0.249	1.525	1.798	2.242	
	Multiple imputation ½	0.006	-0.023	-0.086	0.559	0.664	0.856	
788	Subject mean	0.105	0.025	-0.009	2.200	2.281	2.753	
	Subscale mean	0.001	-0.003	-0.002	3.166	3.316	4.309	
	Subscale ½ mean	0.002	-0.001	-0.003	0.952	0.994	1.297	
	Item mean	0.001	-0.352	-1.041	3.569	4.075	4.751	
	Multiple imputation	0.006	-0.065	-0.189	1.758	1.962	2.369	
	Multiple imputation ½	0.005	-0.021	-0.073	0.693	0.776	0.982	

	Bias	Imprecision	
	Random	Demog	Subscale	Random	Demog	Subscale	
N		-0.000	-0.001	0.001	0.170	0.173	0.275	
52	Subject mean							
	Subscale mean	-0.001	-0.000	0.000	0.179	0.182	0.277	
	Subscale ½ mean	0.000	0.000	0.002	0.143	0.143	0.221	
	Item mean	0.002	-0.044	-0.270	0.362	0.439	1.278	
	Multiple imputation	0.002	0.001	-0.033	0.187	0.208	0.348	
	Multiple imputation ½	0.002	0.002	-0.026	0.161	0.169	0.286	
128	Subject mean	-0.001	0.001	0.001	0.173	0.173	0.267	
	Subscale mean	-0.002	0.001	0.001	0.181	0.183	0.271	
	Subscale ½ mean	0.000	0.002	0.001	0.147	0.150	0.214	
	Item mean	-0.001	-0.046	-0.274	0.370	0.434	1.287	
	Multiple imputation	0.001	0.000	-0.035	0.160	0.173	0.271	
	Multiple imputation ½	0.000	0.000	-0.029	0.135	0.150	0.219	
788	Subject mean	-0.000	0.000	0.000	0.232	0.240	0.352	
	Subscale mean	-0.001	0.000	0.000	0.242	0.248	0.357	
	Subscale ½ mean	-0.001	0.000	-0.000	0.196	0.201	0.290	
	Item mean	-0.001	-0.059	-0.330	0.493	0.565	1.474	
	Multiple imputation	0.001	-0.003	-0.042	0.193	0.211	0.310	
	Multiple imputation ½	0.001	-0.002	-0.036	0.162	0.177	0.257	

	Bias	Imprecision	
	Random	Demog	Subscale	Random	Demog	Subscale	
N		-0.001	-0.001	-0.001	0.738	0.762	1.155	
52	Subject mean							
	Subscale mean	-0.003	-0.002	-0.004	0.815	0.810	1.214	
	Subscale ½ mean	0.000	-0.000	0.002	0.088	0.088	0.134	
	Item mean	0.000	-0.117	-0.699	1.696	1.993	6.498	
	Multiple imputation	0.003	-0.010	-0.144	0.698	0.805	1.356	
	Multiple imputation ½	0.001	-0.001	-0.019	0.098	0.098	0.167	
128	Subject mean	0.003	0.002	-0.001	0.746	0.795	1.154	
	Subscale mean	0.003	-0.001	-0.003	0.814	0.823	1.192	
	Subscale ½ mean	0.001	0.000	0.001	0.092	0.094	0.127	
	Item mean	-0.008	-0.120	-0.707	1.652	1.962	6.573	
	Multiple imputation	0.002	-0.011	-0.142	0.613	0.687	1.170	
	Multiple imputation ½	-0.000	-0.001	-0.019	0.083	0.091	0.130	
788	Subject mean	-0.001	-0.002	-0.000	0.996	1.031	1.540	
	Subscale mean	-0.002	-0.003	-0.000	1.072	1.097	1.611	
	Subscale ½ mean	-0.000	0.000	-0.000	0.121	0.124	0.186	
	Item mean	-0.004	-0.154	-0.859	2.185	2.521	7.536	
	Multiple imputation	-0.000	-0.020	-0.167	0.764	0.834	1.385	
	Multiple imputation ½	-0.000	-0.002	-0.024	0.100	0.107	0.169	

	Bias	Imprecision	
	Random	Demog	Subscale	Random	Demog	Subscale	
N		-0.003	0.000	-0.002	0.342	0.337	0.513	
52	Subject mean							
	Subscale mean	-0.003	0.002	-0.002	0.359	0.356	0.528	
	Subscale ½ mean	-0.001	0.002	0.001	0.293	0.288	0.429	
	Item mean	0.003	-0.075	-0.439	0.751	0.860	1.858	
	Multiple imputation	0.003	0.005	-0.056	0.412	0.416	0.638	
	Multiple imputation ½	0.003	0.004	-0.047	0.349	0.354	0.530	
128	Subject mean	0.002	0.002	0.003	0.362	0.367	0.523	
	Subscale mean	0.001	0.002	0.002	0.377	0.383	0.545	
	Subscale ½ mean	0.002	0.002	0.003	0.306	0.313	0.438	
	Item mean	-0.003	-0.072	-0.448	0.792	0.872	1.874	
	Multiple imputation	0.004	0.002	-0.053	0.337	0.366	0.511	
	Multiple imputation ½	0.003	0.002	-0.043	0.281	0.306	0.418	
788	Subject mean	-0.000	-0.000	-0.000	0.461	0.480	0.649	
	Subscale mean	-0.001	-0.000	-0.000	0.483	0.502	0.676	
	Subscale ½ mean	-0.000	-0.000	-0.000	0.397	0.411	0.555	
	Item mean	0.003	-0.089	-0.522	1.000	1.142	2.086	
	Multiple imputation	0.003	-0.000	-0.062	0.389	0.436	0.558	
	Multiple imputation ½	0.003	0.000	-0.053	0.329	0.367	0.472	

	Bias	Imprecision	
	Random	Demog	Subscale	Random	Demog	Subscale	
N		0.004	-0.002	-0.004	1.538	1.510	2.188	
52	Subject mean							
	Subscale mean	0.003	-0.001	-0.007	1.674	1.644	2.382	
	Subscale ½ mean	0.003	-0.000	0.001	0.197	0.193	0.280	
	Item mean	-0.002	-0.203	-1.168	3.369	3.941	9.637	
	Multiple imputation	0.016	-0.010	-0.243	1.456	1.597	2.531	
	Multiple imputation ½	0.003	0.000	-0.033	0.206	0.235	0.343	
128	Subject mean	-0.005	0.001	-0.003	1.596	1.616	2.262	
	Subscale mean	-0.005	-0.002	-0.006	1.705	1.687	2.408	
	Subscale ½ mean	0.001	-0.001	-0.001	0.200	0.204	0.291	
	Item mean	0.004	-0.196	-1.203	3.505	3.925	9.778	
	Multiple imputation	0.004	-0.012	-0.227	1.313	1.414	2.143	
	Multiple imputation ½	0.003	-0.002	-0.035	0.180	0.199	0.284	
788	Subject mean	0.001	-0.004	-0.001	2.001	2.078	2.808	
	Subscale mean	-0.000	-0.004	-0.001	2.160	2.227	3.022	
	Subscale ½ mean	-0.001	-0.000	-0.000	0.274	0.278	0.382	
	Item mean	0.002	-0.242	-1.421	4.404	5.094	11.032	
	Multiple imputation	0.006	-0.020	-0.246	1.548	1.716	2.350	
	Multiple imputation ½	0.001	-0.000	-0.038	0.227	0.247	0.331	

	Bias	Imprecision	
	Random	Demog	Subscale	Random	Demog	Subscale	
N		-0.008	-0.002	-0.005	0.856	0.882	1.150	
52	Subject mean							
	Subscale mean	-0.006	0.001	-0.003	0.898	0.926	1.240	
	Subscale ½ mean	-0.005	0.003	0.000	0.744	0.763	1.013	
	Item mean	-0.003	-0.179	-0.628	1.927	2.215	2.608	
	Multiple imputation	0.007	0.009	-0.072	1.016	1.149	1.425	
	Multiple imputation ½	0.002	0.011	-0.059	0.877	0.990	1.208	
128	Subject mean	-0.000	0.006	0.000	0.900	0.950	1.201	
	Subscale mean	0.002	0.005	0.001	0.948	0.984	1.277	
	Subscale ½ mean	0.002	0.004	0.002	0.791	0.826	1.063	
	Item mean	0.003	-0.184	-0.621	1.968	2.273	2.610	
	Multiple imputation	0.009	0.008	-0.064	0.855	0.961	1.148	
	Multiple imputation ½	0.008	0.005	-0.054	0.736	0.841	0.995	
788	Subject mean	0.002	0.000	0.001	1.154	1.197	1.446	
	Subscale mean	0.002	-0.000	0.000	1.212	1.257	1.544	
	Subscale ½ mean	0.001	0.001	0.000	1.021	1.059	1.301	
	Item mean	0.001	-0.152	-0.548	2.496	2.737	2.800	
	Multiple imputation	0.009	0.002	-0.060	0.976	1.057	1.210	
	Multiple imputation ½	0.008	0.003	-0.052	0.850	0.918	1.054	

	Bias	Imprecision	
	Random	Demog	Subscale	Random	Demog	Subscale	
N		-0.001	-0.004	-0.011	3.696	3.838	4.982	
52	Subject mean							
	Subscale mean	0.004	-0.004	-0.014	4.004	4.235	5.504	
	Subscale ½ mean	0.003	0.004	0.001	0.641	0.646	0.863	
	Item mean	-0.008	-0.527	-1.881	8.530	10.069	13.934	
	Multiple imputation	0.026	-0.035	-0.342	3.852	4.432	5.464	
	Multiple imputation ½	0.006	0.004	-0.069	0.780	0.859	1.078	
128	Subject mean	0.009	0.005	-0.001	3.901	4.142	5.119	
	Subscale mean	0.012	0.001	-0.005	4.231	4.460	5.628	
	Subscale ½ mean	0.007	-0.003	0.001	0.702	0.730	0.950	
	Item mean	0.005	-0.570	-1.878	8.749	10.367	13.863	
	Multiple imputation	0.026	-0.021	-0.259	3.350	3.808	4.588	
	Multiple imputation ½	0.010	-0.001	-0.053	0.662	0.750	0.955	
788	Subject mean	-0.000	-0.001	0.000	4.981	5.162	6.244	
	Subscale mean	-0.002	-0.003	0.000	5.433	5.627	6.949	
	Subscale ½ mean	0.001	-0.000	-0.003	1.097	1.115	1.375	
	Item mean	-0.002	-0.491	-1.770	11.031	12.243	14.154	
	Multiple imputation	0.009	-0.015	-0.208	3.877	4.196	4.881	
	Multiple imputation ½	0.005	0.004	-0.052	0.915	0.976	1.173	

	Bias	Imprecision	
	Random	Demog	Subscale	Random	Demog	Subscale	
N		-0.006	-0.000	0.024	0.001	0.001	0.003	
52	Subject mean							
	Subscale mean	-0.001	0.000	-0.001	0.001	0.001	0.002	
	Subscale ½ mean	0.001	-0.000	-0.014	0.002	0.002	0.005	
	Item mean	0.002	-0.015	-0.105	0.002	0.003	0.016	
	Multiple imputation	0.001	0.005	0.005	0.002	0.002	0.003	
	Multiple imputation ½	0.003	0.004	-0.008	0.002	0.003	0.005	
	Complete case	0.005	-0.068	-0.445	0.027	0.036	0.245	
128	Subject mean	-0.007	0.001	0.025	0.001	0.001	0.002	
	Subscale mean	-0.000	0.001	0.001	0.001	0.001	0.001	
	Subscale ½ mean	-0.000	-0.002	-0.014	0.001	0.001	0.002	
	Item mean	-0.000	-0.015	-0.106	0.001	0.001	0.013	
	Multiple imputation	0.001	0.003	0.003	0.001	0.001	0.001	
	Multiple imputation ½	0.000	0.001	-0.012	0.001	0.001	0.002	
	Complete case	0.001	-0.069	-0.450	0.013	0.017	0.223	
788	Subject mean	-0.009	0.001	0.032	0.000	0.000	0.001	
	Subscale mean	-0.001	0.000	0.001	0.000	0.000	0.000	
	Subscale ½ mean	-0.001	-0.004	-0.018	0.000	0.000	0.001	
	Item mean	-0.001	-0.021	-0.126	0.000	0.001	0.016	
	Multiple imputation	-0.000	0.003	0.005	0.000	0.000	0.000	
	Multiple imputation ½	-0.001	-0.001	-0.016	0.000	0.000	0.001	
	Complete case	-0.002	-0.090	-0.543	0.003	0.011	0.298	

	Bias	Imprecision	
	Random	Demog	Subscale	Random	Demog	Subscale	
N		-0.015	-0.002	0.058	0.005	0.006	0.012	
52	Subject mean							
	Subscale mean	0.001	-0.000	-0.007	0.007	0.006	0.011	
	Subscale ½ mean	0.002	-0.044	-0.272	0.017	0.023	0.113	
	Item mean	0.001	-0.043	-0.276	0.010	0.014	0.092	
	Multiple imputation	0.003	0.005	-0.002	0.005	0.007	0.010	
	Multiple imputation ½	0.003	-0.042	-0.273	0.017	0.023	0.113	
	Complete case	0.003	-0.092	-0.570	0.038	0.048	0.367	
128	Subject mean	-0.014	0.002	0.060	0.003	0.002	0.007	
	Subscale mean	0.002	-0.002	-0.007	0.003	0.003	0.005	
	Subscale ½ mean	-0.006	-0.039	-0.277	0.009	0.010	0.095	
	Item mean	-0.003	-0.042	-0.279	0.005	0.006	0.084	
	Multiple imputation	0.002	0.005	-0.004	0.002	0.002	0.003	
	Multiple imputation ½	-0.005	-0.037	-0.278	0.009	0.010	0.095	
	Complete case	-0.010	-0.090	-0.579	0.016	0.023	0.353	
788	Subject mean	-0.021	0.001	0.080	0.001	0.001	0.007	
	Subscale mean	-0.001	-0.001	-0.004	0.001	0.001	0.001	
	Subscale ½ mean	-0.002	-0.057	-0.329	0.002	0.005	0.112	
	Item mean	-0.002	-0.055	-0.329	0.001	0.004	0.110	
	Multiple imputation	-0.000	0.004	0.003	0.001	0.000	0.001	
	Multiple imputation ½	-0.001	-0.055	-0.328	0.002	0.005	0.111	
	Complete case	-0.004	-0.116	-0.703	0.005	0.016	0.498	

	Bias	Imprecision	
	Random	Demog	Subscale	Random	Demog	Subscale	
N		-0.013	-0.001	0.032	0.003	0.003	0.005	
52	Subject mean							
	Subscale mean	0.001	0.001	-0.002	0.003	0.003	0.004	
	Subscale ½ mean	0.001	0.000	-0.023	0.005	0.004	0.008	
	Item mean	0.002	-0.025	-0.174	0.005	0.006	0.037	
	Multiple imputation	0.003	0.008	0.005	0.003	0.004	0.005	
	Multiple imputation ½	0.003	0.005	-0.019	0.005	0.005	0.008	
	Complete case	0.011	-0.116	-0.778	0.063	0.081	0.684	
128	Subject mean	-0.012	-0.000	0.036	0.001	0.001	0.003	
	Subscale mean	0.002	0.002	0.001	0.001	0.001	0.002	
	Subscale ½ mean	0.000	-0.001	-0.025	0.002	0.002	0.004	
	Item mean	-0.001	-0.024	-0.177	0.002	0.003	0.034	
	Multiple imputation	0.003	0.005	0.005	0.001	0.001	0.002	
	Multiple imputation ½	0.001	0.002	-0.021	0.002	0.002	0.003	
	Complete case	-0.007	-0.117	-0.799	0.029	0.040	0.674	
788	Subject mean	-0.017	-0.000	0.037	0.001	0.000	0.002	
	Subscale mean	0.000	0.000	0.001	0.000	0.000	0.000	
	Subscale ½ mean	0.000	-0.005	-0.031	0.001	0.001	0.002	
	Item mean	0.002	-0.028	-0.208	0.001	0.001	0.044	
	Multiple imputation	0.002	0.007	0.003	0.000	0.000	0.000	
	Multiple imputation ½	0.001	0.001	-0.029	0.001	0.001	0.002	
	Complete case	0.007	-0.131	-0.985	0.007	0.023	0.977	

	Bias	Imprecision	
	Random	Demog	Subscale	Random	Demog	Subscale	
N		-0.030	-0.004	0.078	0.013	0.011	0.023	
52	Subject mean							
	Subscale mean	0.003	0.002	-0.016	0.015	0.012	0.019	
	Subscale ½ mean	-0.003	-0.072	-0.463	0.041	0.048	0.280	
	Item mean	-0.001	-0.070	-0.467	0.023	0.028	0.243	
	Multiple imputation	0.010	0.016	-0.007	0.012	0.014	0.018	
	Multiple imputation ½	0.000	-0.067	-0.461	0.042	0.048	0.277	
	Complete case	-0.004	-0.161	-1.018	0.085	0.111	1.111	
128	Subject mean	-0.035	-0.003	0.087	0.006	0.005	0.014	
	Subscale mean	-0.001	-0.002	-0.008	0.006	0.006	0.008	
	Subscale ½ mean	0.006	-0.063	-0.473	0.019	0.022	0.255	
	Item mean	0.002	-0.066	-0.475	0.010	0.014	0.236	
	Multiple imputation	0.002	0.012	-0.001	0.005	0.005	0.006	
	Multiple imputation ½	0.006	-0.060	-0.473	0.019	0.022	0.254	
	Complete case	0.004	-0.152	-1.058	0.039	0.058	1.151	
788	Subject mean	-0.040	-0.001	0.090	0.003	0.001	0.010	
	Subscale mean	0.000	-0.002	-0.006	0.002	0.002	0.002	
	Subscale ½ mean	0.001	-0.080	-0.568	0.005	0.011	0.328	
	Item mean	0.001	-0.077	-0.568	0.003	0.008	0.325	
	Multiple imputation	0.003	0.016	-0.004	0.001	0.001	0.002	
	Multiple imputation ½	0.002	-0.073	-0.569	0.005	0.010	0.329	
	Complete case	0.002	-0.177	-1.326	0.011	0.037	1.763	

	Bias	Imprecision	
	Random	Demog	Subscale	Random	Demog	Subscale	
N		-0.035	-0.005	0.020	0.009	0.007	0.010	
52	Subject mean							
	Subscale mean	-0.004	0.000	-0.001	0.008	0.007	0.010	
	Subscale ½ mean	-0.001	-0.003	-0.036	0.012	0.011	0.016	
	Item mean	-0.001	-0.056	-0.256	0.013	0.016	0.079	
	Multiple imputation	0.002	0.018	0.001	0.009	0.010	0.012	
	Multiple imputation ½	0.004	0.014	-0.034	0.013	0.015	0.017	
	Complete case	0.007	-0.328	-1.484	0.231	0.328	2.438	
128	Subject mean	-0.034	0.003	0.022	0.004	0.003	0.004	
	Subscale mean	0.001	0.004	0.002	0.003	0.003	0.004	
	Subscale ½ mean	0.001	-0.003	-0.035	0.005	0.006	0.008	
	Item mean	0.001	-0.059	-0.253	0.006	0.009	0.069	
	Multiple imputation	0.003	0.020	0.000	0.003	0.004	0.004	
	Multiple imputation ½	0.003	0.009	-0.035	0.005	0.006	0.007	
	Complete case	0.001	-0.369	-1.507	0.102	0.226	2.370	
788	Subject mean	-0.040	-0.010	0.004	0.002	0.001	0.001	
	Subscale mean	0.002	0.001	0.002	0.001	0.001	0.001	
	Subscale ½ mean	0.000	-0.006	-0.031	0.001	0.002	0.002	
	Item mean	0.000	-0.043	-0.225	0.001	0.003	0.052	
	Multiple imputation	0.004	0.016	-0.002	0.001	0.001	0.001	
	Multiple imputation ½	0.002	0.008	-0.034	0.001	0.001	0.003	
	Complete case	-0.005	-0.304	-1.606	0.033	0.118	2.606	

	Bias	Imprecision	
	Random	Demog	Subscale	Random	Demog	Subscale	
N		-0.085	-0.007	0.050	0.034	0.027	0.041	
52	Subject mean							
	Subscale mean	-0.002	0.003	-0.015	0.036	0.033	0.044	
	Subscale ½ mean	-0.000	-0.173	-0.771	0.134	0.161	0.744	
	Item mean	-0.009	-0.167	-0.771	0.061	0.089	0.648	
	Multiple imputation	0.010	0.052	-0.024	0.034	0.040	0.052	
	Multiple imputation ½	0.002	-0.152	-0.778	0.131	0.157	0.747	
	Complete case	-0.028	-0.492	-2.196	0.329	0.559	5.049	
128	Subject mean	-0.076	0.001	0.054	0.018	0.014	0.017	
	Subscale mean	0.010	0.002	-0.006	0.016	0.015	0.019	
	Subscale ½ mean	0.005	-0.181	-0.760	0.058	0.090	0.648	
	Item mean	0.005	-0.181	-0.765	0.026	0.055	0.606	
	Multiple imputation	0.017	0.053	-0.011	0.013	0.016	0.017	
	Multiple imputation ½	0.008	-0.162	-0.767	0.057	0.081	0.654	
	Complete case	-0.001	-0.561	-2.298	0.152	0.440	5.369	
788	Subject mean	-0.105	-0.026	0.009	0.014	0.004	0.004	
	Subscale mean	-0.002	-0.001	-0.005	0.004	0.004	0.005	
	Subscale ½ mean	-0.001	-0.143	-0.728	0.016	0.035	0.545	
	Item mean	-0.003	-0.140	-0.728	0.007	0.025	0.535	
	Multiple imputation	0.003	0.050	-0.019	0.003	0.006	0.004	
	Multiple imputation ½	0.001	-0.120	-0.738	0.015	0.028	0.558	
	Complete case	-0.003	-0.525	-2.776	0.062	0.308	7.727	

	Bias	Imprecision	
	Random	Demog	Subscale	Random	Demog	Subscale	
N		0.006	-0.000	-0.023	0.002	0.002	0.003	
52	Subject mean							
	Subscale mean	-0.000	-0.000	0.001	0.002	0.002	0.003	
	Subscale ½ mean	0.000	-0.009	-0.034	0.003	0.004	0.010	
	Item mean	0.000	-0.029	-0.164	0.003	0.004	0.033	
	Multiple imputation	0.001	-0.004	-0.038	0.002	0.002	0.005	
	Multiple imputation ½	0.001	-0.011	-0.068	0.003	0.004	0.014	
	Complete case	-0.000	-0.119	-0.679	0.026	0.043	0.500	
128	Subject mean	0.006	-0.000	-0.024	0.001	0.001	0.002	
	Subscale mean	-0.001	0.000	-0.000	0.001	0.001	0.001	
	Subscale ½ mean	0.000	-0.006	-0.032	0.001	0.001	0.004	
	Item mean	-0.001	-0.030	-0.167	0.001	0.002	0.031	
	Multiple imputation	0.000	-0.003	-0.038	0.001	0.001	0.003	
	Multiple imputation ½	0.000	-0.009	-0.065	0.001	0.001	0.008	
	Complete case	-0.003	-0.126	-0.694	0.012	0.027	0.500	
788	Subject mean	0.009	-0.000	-0.032	0.000	0.000	0.001	
	Subscale mean	-0.000	-0.000	-0.001	0.000	0.000	0.000	
	Subscale ½ mean	0.000	-0.006	-0.036	0.000	0.000	0.002	
	Item mean	0.000	-0.038	-0.204	0.000	0.002	0.042	
	Multiple imputation	0.001	-0.006	-0.046	0.000	0.000	0.002	
	Multiple imputation ½	0.001	-0.011	-0.075	0.000	0.000	0.006	
	Complete case	-0.000	-0.161	-0.871	0.003	0.028	0.763	

	Bias	Imprecision	
	Random	Demog	Subscale	Random	Demog	Subscale	
N		0.015	0.001	-0.059	0.006	0.007	0.013	
52	Subject mean							
	Subscale mean	-0.005	-0.003	-0.010	0.009	0.010	0.014	
	Subscale ½ mean	0.001	-0.072	-0.420	0.020	0.026	0.228	
	Item mean	-0.000	-0.074	-0.423	0.011	0.017	0.190	
	Multiple imputation	0.000	-0.015	-0.142	0.007	0.008	0.032	
	Multiple imputation ½	0.000	-0.075	-0.463	0.020	0.026	0.258	
	Complete case	0.001	-0.152	-0.875	0.037	0.059	0.782	
128	Subject mean	0.017	-0.000	-0.061	0.003	0.003	0.008	
	Subscale mean	0.001	-0.001	-0.008	0.004	0.005	0.006	
	Subscale ½ mean	-0.003	-0.079	-0.423	0.008	0.015	0.200	
	Item mean	-0.005	-0.079	-0.429	0.005	0.011	0.189	
	Multiple imputation	0.001	-0.017	-0.137	0.003	0.003	0.023	
	Multiple imputation ½	-0.004	-0.083	-0.463	0.008	0.015	0.232	
	Complete case	-0.010	-0.161	-0.893	0.015	0.040	0.804	
788	Subject mean	0.020	-0.003	-0.080	0.001	0.001	0.007	
	Subscale mean	-0.001	-0.003	-0.009	0.001	0.001	0.002	
	Subscale ½ mean	-0.002	-0.097	-0.528	0.002	0.011	0.284	
	Item mean	-0.001	-0.098	-0.530	0.001	0.011	0.282	
	Multiple imputation	0.000	-0.024	-0.170	0.001	0.001	0.030	
	Multiple imputation ½	-0.001	-0.103	-0.577	0.002	0.012	0.338	
	Complete case	-0.004	-0.209	-1.127	0.005	0.046	1.271	

	Bias	Imprecision	
	Random	Demog	Subscale	Random	Demog	Subscale	
N		0.010	0.002	-0.034	0.004	0.004	0.007	
52	Subject mean							
	Subscale mean	-0.004	0.000	-0.001	0.004	0.004	0.006	
	Subscale ½ mean	-0.002	-0.011	-0.050	0.005	0.007	0.016	
	Item mean	0.001	-0.050	-0.266	0.006	0.009	0.080	
	Multiple imputation	-0.000	-0.003	-0.061	0.004	0.005	0.011	
	Multiple imputation ½	-0.000	-0.014	-0.103	0.006	0.008	0.025	
	Complete case	0.014	-0.219	-1.170	0.059	0.114	1.448	
128	Subject mean	0.014	0.002	-0.034	0.002	0.002	0.004	
	Subscale mean	-0.001	0.000	0.001	0.002	0.002	0.003	
	Subscale ½ mean	-0.001	-0.008	-0.049	0.003	0.003	0.007	
	Item mean	-0.001	-0.048	-0.272	0.002	0.005	0.078	
	Multiple imputation	0.001	-0.003	-0.058	0.001	0.002	0.006	
	Multiple imputation ½	-0.000	-0.012	-0.099	0.003	0.003	0.015	
	Complete case	-0.003	-0.218	-1.218	0.026	0.072	1.518	
788	Subject mean	0.016	-0.000	-0.037	0.001	0.000	0.002	
	Subscale mean	-0.001	-0.000	-0.002	0.000	0.001	0.001	
	Subscale ½ mean	0.001	-0.010	-0.053	0.001	0.001	0.004	
	Item mean	0.002	-0.061	-0.314	0.001	0.004	0.099	
	Multiple imputation	0.001	-0.007	-0.064	0.000	0.000	0.005	
	Multiple imputation ½	0.003	-0.015	-0.109	0.001	0.001	0.013	
	Complete case	0.006	-0.287	-1.476	0.007	0.088	2.186	

	Bias	Imprecision	
	Random	Demog	Subscale	Random	Demog	Subscale	
N		0.034	0.003	-0.082	0.014	0.014	0.025	
52	Subject mean							
	Subscale mean	0.000	-0.006	-0.010	0.018	0.020	0.028	
	Subscale ½ mean	0.002	-0.134	-0.701	0.041	0.064	0.575	
	Item mean	-0.002	-0.132	-0.702	0.022	0.041	0.514	
	Multiple imputation	0.005	-0.027	-0.236	0.013	0.016	0.082	
	Multiple imputation ½	-0.000	-0.139	-0.774	0.040	0.064	0.672	
	Complete case	-0.003	-0.285	-1.540	0.077	0.161	2.409	
128	Subject mean	0.031	0.003	-0.090	0.007	0.006	0.017	
	Subscale mean	-0.004	-0.003	-0.016	0.008	0.009	0.014	
	Subscale ½ mean	-0.001	-0.130	-0.722	0.018	0.035	0.557	
	Item mean	0.002	-0.130	-0.728	0.011	0.026	0.540	
	Multiple imputation	0.002	-0.024	-0.227	0.005	0.006	0.062	
	Multiple imputation ½	0.002	-0.135	-0.790	0.018	0.036	0.656	
	Complete case	0.010	-0.288	-1.614	0.037	0.113	2.623	
788	Subject mean	0.042	-0.002	-0.092	0.003	0.001	0.010	
	Subscale mean	-0.001	-0.006	-0.014	0.002	0.002	0.003	
	Subscale ½ mean	0.002	-0.163	-0.851	0.005	0.031	0.734	
	Item mean	0.001	-0.165	-0.853	0.003	0.029	0.730	
	Multiple imputation	0.003	-0.035	-0.242	0.001	0.003	0.061	
	Multiple imputation ½	0.004	-0.170	-0.924	0.005	0.033	0.863	
	Complete case	0.003	-0.383	-1.981	0.010	0.152	3.927	

	Bias	Imprecision	
	Random	Demog	Subscale	Random	Demog	Subscale	
N		0.028	0.002	-0.025	0.010	0.009	0.013	
52	Subject mean							
	Subscale mean	-0.003	0.001	-0.002	0.010	0.010	0.015	
	Subscale ½ mean	-0.000	-0.025	-0.067	0.016	0.019	0.026	
	Item mean	-0.002	-0.123	-0.372	0.014	0.030	0.154	
	Multiple imputation	0.005	-0.010	-0.074	0.011	0.012	0.023	
	Multiple imputation ½	0.003	-0.033	-0.130	0.016	0.021	0.040	
	Complete case	-0.000	-0.695	-2.122	0.201	0.699	4.720	
128	Subject mean	0.034	0.004	-0.022	0.005	0.004	0.006	
	Subscale mean	0.001	0.001	-0.001	0.004	0.005	0.006	
	Subscale ½ mean	0.000	-0.020	-0.062	0.006	0.008	0.012	
	Item mean	0.002	-0.125	-0.368	0.006	0.022	0.142	
	Multiple imputation	0.006	-0.012	-0.065	0.004	0.005	0.010	
	Multiple imputation ½	0.004	-0.030	-0.118	0.006	0.008	0.022	
	Complete case	0.011	-0.761	-2.182	0.095	0.674	4.857	
788	Subject mean	0.043	0.010	-0.004	0.003	0.001	0.001	
	Subscale mean	0.000	-0.001	-0.002	0.001	0.001	0.002	
	Subscale ½ mean	0.001	-0.017	-0.052	0.002	0.002	0.005	
	Item mean	0.001	-0.110	-0.323	0.001	0.013	0.106	
	Multiple imputation	0.005	-0.014	-0.058	0.001	0.001	0.005	
	Multiple imputation ½	0.006	-0.028	-0.101	0.001	0.002	0.012	
	Complete case	-0.004	-0.776	-2.291	0.028	0.628	5.273	

	Bias	Imprecision	
	Random	Demog	Subscale	Random	Demog	Subscale	
N		0.083	0.002	-0.061	0.037	0.033	0.047	
52	Subject mean							
	Subscale mean	0.004	-0.011	-0.024	0.046	0.051	0.071	
	Subscale ½ mean	0.000	-0.357	-1.102	0.141	0.258	1.377	
	Item mean	0.001	-0.360	-1.110	0.062	0.189	1.277	
	Multiple imputation	0.016	-0.086	-0.318	0.038	0.055	0.173	
	Multiple imputation ½	0.005	-0.382	-1.218	0.136	0.274	1.638	
	Complete case	-0.016	-1.026	-3.168	0.348	1.347	10.162	
128	Subject mean	0.085	0.004	-0.056	0.020	0.015	0.023	
	Subscale mean	0.003	-0.007	-0.021	0.020	0.024	0.032	
	Subscale ½ mean	0.001	-0.385	-1.107	0.056	0.210	1.297	
	Item mean	-0.001	-0.389	-1.113	0.025	0.175	1.261	
	Multiple imputation	0.009	-0.074	-0.249	0.014	0.025	0.088	
	Multiple imputation ½	0.006	-0.403	-1.192	0.054	0.221	1.488	
	Complete case	-0.005	-1.161	-3.323	0.141	1.462	11.101	
788	Subject mean	0.105	0.025	-0.009	0.014	0.004	0.004	
	Subscale mean	0.001	-0.007	-0.014	0.006	0.006	0.007	
	Subscale ½ mean	-0.002	-0.348	-1.040	0.017	0.137	1.099	
	Item mean	0.001	-0.352	-1.041	0.007	0.129	1.089	
	Multiple imputation	0.006	-0.065	-0.189	0.004	0.008	0.041	
	Multiple imputation ½	0.002	-0.368	-1.109	0.016	0.149	1.247	
	Complete case	-0.002	-1.338	-3.953	0.054	1.815	15.638	

	Bias	Imprecision	
	Random	Demog	Subscale	Random	Demog	Subscale	
N		-0.000	-0.001	0.001	0.003	0.003	0.006	
52	Subject mean							
	Subscale mean	-0.001	-0.000	0.000	0.003	0.003	0.006	
	Subscale ½ mean	0.003	-0.016	-0.091	0.010	0.014	0.040	
	Item mean	0.002	-0.044	-0.270	0.007	0.010	0.084	
	Multiple imputation	0.002	0.001	-0.033	0.004	0.004	0.008	
	Multiple imputation ½	0.004	-0.015	-0.118	0.010	0.014	0.046	
	Complete case	-0.001	-0.232	-1.377	0.109	0.174	1.995	
128	Subject mean	-0.001	0.001	0.001	0.001	0.001	0.002	
	Subscale mean	-0.002	0.001	0.001	0.001	0.001	0.002	
	Subscale ½ mean	0.001	-0.015	-0.090	0.004	0.005	0.021	
	Item mean	-0.001	-0.046	-0.274	0.003	0.005	0.080	
	Multiple imputation	0.001	0.000	-0.035	0.001	0.001	0.003	
	Multiple imputation ½	0.001	-0.016	-0.120	0.004	0.005	0.027	
	Complete case	-0.003	-0.240	-1.409	0.053	0.105	2.029	
788	Subject mean	-0.000	0.000	0.000	0.000	0.000	0.001	
	Subscale mean	-0.001	0.000	0.000	0.000	0.000	0.001	
	Subscale ½ mean	-0.001	-0.020	-0.107	0.001	0.002	0.015	
	Item mean	-0.001	-0.059	-0.330	0.001	0.004	0.110	
	Multiple imputation	0.001	-0.003	-0.042	0.000	0.000	0.002	
	Multiple imputation ½	0.001	-0.022	-0.143	0.001	0.002	0.023	
	Complete case	-0.001	-0.310	-1.754	0.013	0.105	3.084	

	Bias	Imprecision	
	Random	Demog	Subscale	Random	Demog	Subscale	
N		-0.000	-0.001	-0.001	0.014	0.015	0.023	
52	Subject mean							
	Subscale mean	-0.003	-0.005	-0.028	0.017	0.018	0.032	
	Subscale ½ mean	0.008	-0.180	-1.067	0.093	0.133	1.291	
	Item mean	0.000	-0.117	-0.699	0.033	0.049	0.522	
	Multiple imputation	0.003	-0.010	-0.144	0.014	0.015	0.045	
	Multiple imputation ½	0.008	-0.180	-1.088	0.094	0.133	1.326	
	Complete case	0.004	-0.245	-1.454	0.126	0.187	2.197	
128	Subject mean	0.002	0.002	-0.002	0.006	0.006	0.009	
	Subscale mean	0.004	-0.005	-0.026	0.008	0.008	0.013	
	Subscale ½ mean	-0.014	-0.180	-1.081	0.040	0.072	1.236	
	Item mean	-0.008	-0.120	-0.707	0.014	0.027	0.515	
	Multiple imputation	0.002	-0.012	-0.142	0.005	0.006	0.029	
	Multiple imputation ½	-0.015	-0.181	-1.101	0.040	0.072	1.275	
	Complete case	-0.021	-0.254	-1.483	0.051	0.113	2.235	
788	Subject mean	-0.001	-0.002	-0.000	0.002	0.002	0.003	
	Subscale mean	-0.002	-0.006	-0.026	0.002	0.002	0.004	
	Subscale ½ mean	-0.005	-0.239	-1.331	0.011	0.065	1.786	
	Item mean	-0.004	-0.154	-0.859	0.004	0.026	0.742	
	Multiple imputation	-0.000	-0.020	-0.167	0.001	0.002	0.030	
	Multiple imputation ½	-0.005	-0.241	-1.355	0.011	0.066	1.850	
	Complete case	-0.007	-0.327	-1.846	0.016	0.116	3.413	

	Bias	Imprecision	
	Random	Demog	Subscale	Random	Demog	Subscale	
N		-0.003	0.000	-0.002	0.006	0.006	0.010	
52	Subject mean							
	Subscale mean	-0.003	0.002	-0.002	0.007	0.007	0.011	
	Subscale ½ mean	-0.002	-0.022	-0.139	0.021	0.024	0.065	
	Item mean	0.003	-0.075	-0.439	0.014	0.021	0.211	
	Multiple imputation	0.003	0.005	-0.056	0.008	0.008	0.016	
	Multiple imputation ½	0.003	-0.020	-0.186	0.022	0.026	0.082	
	Complete case	0.023	-0.421	-2.437	0.258	0.441	6.123	
128	Subject mean	0.002	0.002	0.003	0.003	0.003	0.004	
	Subscale mean	0.001	0.002	0.002	0.003	0.003	0.005	
	Subscale ½ mean	-0.003	-0.022	-0.146	0.009	0.010	0.041	
	Item mean	-0.003	-0.072	-0.448	0.006	0.011	0.208	
	Multiple imputation	0.004	0.002	-0.053	0.003	0.003	0.007	
	Multiple imputation ½	-0.001	-0.023	-0.191	0.009	0.010	0.056	
	Complete case	-0.011	-0.419	-2.545	0.115	0.277	6.557	
788	Subject mean	-0.000	-0.000	-0.000	0.001	0.001	0.001	
	Subscale mean	-0.001	-0.000	-0.001	0.001	0.001	0.001	
	Subscale ½ mean	0.002	-0.029	-0.166	0.002	0.004	0.032	
	Item mean	0.003	-0.089	-0.522	0.001	0.009	0.274	
	Multiple imputation	0.003	-0.000	-0.062	0.001	0.001	0.005	
	Multiple imputation ½	0.005	-0.029	-0.218	0.002	0.003	0.052	
	Complete case	0.017	-0.531	-3.148	0.032	0.302	9.925	

	Bias	Imprecision	
	Random	Demog	Subscale	Random	Demog	Subscale	
N		0.004	-0.002	-0.004	0.029	0.030	0.045	
52	Subject mean							
	Subscale mean	0.003	-0.007	-0.044	0.035	0.036	0.058	
	Subscale ½ mean	-0.008	-0.322	-1.835	0.205	0.320	3.646	
	Item mean	-0.002	-0.203	-1.168	0.068	0.110	1.423	
	Multiple imputation	0.016	-0.010	-0.244	0.028	0.034	0.109	
	Multiple imputation ½	-0.007	-0.321	-1.869	0.203	0.319	3.756	
	Complete case	-0.008	-0.447	-2.580	0.274	0.474	6.811	
128	Subject mean	-0.005	0.001	-0.003	0.013	0.014	0.019	
	Subscale mean	-0.005	-0.008	-0.043	0.015	0.016	0.026	
	Subscale ½ mean	0.010	-0.307	-1.901	0.101	0.176	3.740	
	Item mean	0.004	-0.196	-1.203	0.032	0.065	1.472	
	Multiple imputation	0.004	-0.012	-0.227	0.011	0.012	0.071	
	Multiple imputation ½	0.012	-0.308	-1.935	0.100	0.176	3.862	
	Complete case	0.015	-0.445	-2.697	0.130	0.304	7.338	
788	Subject mean	0.001	-0.004	-0.001	0.003	0.003	0.005	
	Subscale mean	-0.001	-0.010	-0.040	0.004	0.004	0.007	
	Subscale ½ mean	0.002	-0.391	-2.302	0.024	0.171	5.327	
	Item mean	0.002	-0.242	-1.421	0.008	0.064	2.024	
	Multiple imputation	0.006	-0.020	-0.247	0.003	0.003	0.065	
	Multiple imputation ½	0.004	-0.391	-2.340	0.024	0.171	5.501	
	Complete case	0.005	-0.565	-3.340	0.035	0.339	11.166	

	Bias	Imprecision	
	Random	Demog	Subscale	Random	Demog	Subscale	
N		-0.008	-0.002	-0.005	0.017	0.016	0.024	
52	Subject mean							
	Subscale mean	-0.006	0.001	-0.003	0.018	0.017	0.025	
	Subscale ½ mean	0.002	-0.052	-0.196	0.056	0.066	0.106	
	Item mean	-0.003	-0.179	-0.628	0.033	0.066	0.425	
	Multiple imputation	0.007	0.009	-0.072	0.020	0.022	0.034	
	Multiple imputation ½	0.009	-0.043	-0.256	0.057	0.071	0.136	
	Complete case	-0.000	-1.414	-4.992	0.993	3.004	25.532	
128	Subject mean	-0.000	0.006	0.000	0.007	0.008	0.010	
	Subscale mean	0.002	0.005	0.001	0.008	0.008	0.010	
	Subscale ½ mean	0.002	-0.054	-0.190	0.024	0.029	0.067	
	Item mean	0.003	-0.184	-0.621	0.014	0.047	0.398	
	Multiple imputation	0.009	0.008	-0.064	0.007	0.008	0.014	
	Multiple imputation ½	0.007	-0.053	-0.246	0.023	0.029	0.091	
	Complete case	0.026	-1.590	-5.213	0.487	2.909	27.457	
788	Subject mean	0.002	0.000	0.001	0.002	0.002	0.003	
	Subscale mean	0.002	-0.000	0.000	0.002	0.002	0.003	
	Subscale ½ mean	-0.000	-0.045	-0.164	0.006	0.009	0.034	
	Item mean	0.001	-0.152	-0.548	0.003	0.026	0.304	
	Multiple imputation	0.009	0.002	-0.060	0.002	0.002	0.006	
	Multiple imputation ½	0.007	-0.043	-0.217	0.005	0.008	0.053	
	Complete case	-0.011	-1.678	-6.081	0.154	2.914	37.059	

	Bias	Imprecision	
	Random	Demog	Subscale	Random	Demog	Subscale	
N		-0.001	-0.004	-0.011	0.072	0.073	0.107	
52	Subject mean							
	Subscale mean	0.004	-0.016	-0.064	0.091	0.093	0.133	
	Subscale ½ mean	-0.010	-0.943	-3.323	0.773	1.670	11.808	
	Item mean	-0.008	-0.527	-1.881	0.187	0.456	3.653	
	Multiple imputation	0.026	-0.035	-0.342	0.082	0.095	0.245	
	Multiple imputation ½	-0.007	-0.943	-3.393	0.759	1.663	12.235	
	Complete case	-0.042	-1.535	-5.441	1.168	3.401	30.089	
128	Subject mean	0.009	0.004	-0.002	0.032	0.036	0.042	
	Subscale mean	0.013	-0.012	-0.052	0.040	0.042	0.054	
	Subscale ½ mean	0.013	-1.024	-3.380	0.301	1.345	11.768	
	Item mean	0.005	-0.570	-1.878	0.071	0.386	3.576	
	Multiple imputation	0.026	-0.021	-0.259	0.031	0.036	0.113	
	Multiple imputation ½	0.016	-1.022	-3.433	0.294	1.335	12.118	
	Complete case	-0.007	-1.749	-5.705	0.495	3.452	32.754	
788	Subject mean	-0.000	-0.001	-0.000	0.008	0.009	0.011	
	Subscale mean	-0.001	-0.015	-0.038	0.010	0.011	0.014	
	Subscale ½ mean	-0.003	-0.975	-3.511	0.104	1.029	12.422	
	Item mean	-0.002	-0.491	-1.770	0.019	0.254	3.142	
	Multiple imputation	0.009	-0.015	-0.208	0.007	0.008	0.052	
	Multiple imputation ½	0.001	-0.971	-3.561	0.101	1.018	12.767	
	Complete case	-0.005	-1.903	-6.875	0.197	3.715	47.314	
