# Supplementary material for: Handling missing items in the Hospital Anxiety and Depression Scale (HADS): a simulation study
Source: BMC Res Notes. 2016 Oct 22;9:479. doi: 10.1186/s13104-016-2284-z (PMC5075158; doi:10.1186/s13104-016-2284-z)
Supplement: Supplementary file 2 — Additional file 2: Appendix S2. Simulation increasing number of missing items. [file 13104_2016_2284_MOESM2_ESM.docx]

Web Appendix B. Simulation increasing number of missing items.

To further investigate the effect of high numbers of missing items within an individual, we conducted another small simulation study to compare the subscale mean and the subscale half mean methods for population measures. We let p_item_ range from 0.5 to 0.929, which corresponds to 7 to 13 missing items out of the 14. We used p_sub_ = 0.1 and 0.5 (= probability a subject has a missing item) and 1000 simulated datasets of n=52 with subscale dependent missingness mechanism. We found that when p_sub_ = 0.1, both methods worked well for bias, even with high numbers of missing items, although coverage was poor for the subscale half mean past 9 or 10 missing items. When psub = 0.5 the subscale mean performed well, in terms of bias, Imprecision and coverage, for up to 12 missing items. The half mean method broke down much sooner. For example, with 9 missing items, the bias for the subscale mean was -0.10, as compared to -2.19 for the half method. This indicates that very few complete items may be needed, if inference is population based.

| **Anxiety** |  |  | **p_sub_ = 0.1** |  |  | **p_sub_ = 0.5** |  |
| --- | --- | --- | --- | --- | --- | --- | --- |
| **P_item_ (number missing items)** | **Method** | **Bias** | **Imprecision** | **Coverage** | **Bias** | **Imprecision** | **Coverage** |
| 0.5 (7) | Subscale mean | -0.02 | 0.01 | 100 | -0.01 | 0.05 | 100 |
|  | Subscale ½ mean | -0.43 | 0.22 | 99 | -1.06 | 1.26 | 70 |
| 0.571 (8) | Subscale mean | -0.01 | 0.02 | 100 | -0.03 | 0.06 | 100 |
|  | Subscale ½ mean | -0.53 | 0.32 | 99 | -1.63 | 2.85 | 22 |
| 0.643 (9) | Subscale mean | -0.05 | 0.02 | 100 | -0.08 | 0.08 | 100 |
|  | Subscale ½ mean | -0.68 | 0.51 | 87 | -2.09 | 4.60 | 5 |
| 0.714 (10) | Subscale mean | -0.07 | 0.03 | 100 | -0.16 | 0.14 | 99 |
|  | Subscale ½ mean | -0.77 | 0.63 | 84 | -2.63 | 7.14 | 0 |
| 0.786 (11) | Subscale mean | -0.15 | 0.07 | 99 | -0.45 | 0.35 | 97 |
|  | Subscale ½ mean | -0.85 | 0.74 | 80 | -3.00 | 9.15 | 0 |
| 0.875 (12) | Subscale mean | -0.26 | 0.11 | 100 | -0.71 | 0.70 | 88 |
|  | Subscale ½ mean | -0.86 | 0.75 | 79 | -3.17 | 10.19 | 0 |
| 0.929 (13) | Subscale mean | -0.48 | 0.28 | 99 | -1.40 | 2.28 | 51 |
|  | Subscale ½ mean | -0.86 | 0.76 | 82 | -3.20 | 10.36 | 0 |

| **Depression** |  |  | **p_sub_ = 0.1** |  |  | **p_sub_ = 0.5** |  |
| --- | --- | --- | --- | --- | --- | --- | --- |
| **P_item_ (number missing items)** | **Method** | **Bias** | **Imprecision** | **Coverage** | **Bias** | **Imprecision** | **Coverage** |
| 0.5 (7) | Subscale mean | -0.01 | 0.01 | 100 | 0.01 | 0.05 | 100 |
|  | Subscale ½ mean | -0.31 | 0.13 | 100 | -0.68 | 0.62 | 90 |
| 0.571 (8) | Subscale mean | -0.03 | 0.02 | 100 | -0.03 | 0.08 | 100 |
|  | Subscale ½ mean | -0.36 | 0.16 | 100 | -1.03 | 1.23 | 71 |
| 0.643 (9) | Subscale mean | -0.01 | 0.02 | 100 | -0.04 | 0.12 | 99 |
|  | Subscale ½ mean | -0.47 | 0.26 | 99 | -1.35 | 2.07 | 43 |
| 0.714 (10) | Subscale mean | -0.05 | 0.03 | 100 | -0.09 | 0.13 | 100 |
|  | Subscale ½ mean | -0.53 | 0.31 | 98 | -1.64 | 2.93 | 24 |
| 0.786 (11) | Subscale mean | -0.11 | 0.05 | 100 | -0.30 | 0.27 | 100 |
|  | Subscale ½ mean | -0.56 | 0.35 | 99 | -1.91 | 3.83 | 11 |
| 0.875 (12) | Subscale mean | -0.18 | 0.08 | 100 | -0.39 | 0.38 | 97 |
|  | Subscale ½ mean | -0.57 | 0.36 | 99 | -1.99 | 4.20 | 10 |
| 0.929 (13) | Subscale mean | -0.33 | 0.16 | 100 | -0.90 | 1.12 | 76 |
|  | Subscale ½ mean | -0.58 | 0.37 | 99 | -2.05 | 4.40 | 3 |

| **Distress** |  |  | **p_sub_ = 0.1** |  |  | **p_sub_ = 0.5** |  |
| --- | --- | --- | --- | --- | --- | --- | --- |
| **P_item_ (number missing items)** | **Method** | **Bias** | **Imprecision** | **Coverage** | **Bias** | **Imprecision** | **Coverage** |
| 0.5 (7) | Subscale mean | -0.04 | 0.03 | 100 | -0.02 | 0.10 | 100 |
|  | Subscale ½ mean | -1.11 | 1.35 | 97 | -3.23 | 11.05 | 18 |
| 0.571 (8) | Subscale mean | -0.07 | 0.05 | 100 | -0.13 | 0.17 | 100 |
|  | Subscale ½ mean | -1.23 | 1.62 | 95 | -4.24 | 18.53 | 1 |
| 0.643 (9) | Subscale mean | -0.12 | 0.08 | 100 | -0.26 | 0.29 | 100 |
|  | Subscale ½ mean | -1.43 | 2.14 | 88 | -4.79 | 23.47 | 0 |
| 0.714 (10) | Subscale mean | -0.23 | 0.14 | 100 | -0.53 | 0.66 | 100 |
|  | Subscale ½ mean | -1.47 | 2.22 | 85 | -5.05 | 26.11 | 0 |
| 0.786 (11) | Subscale mean | -0.48 | 0.36 | 100 | -1.30 | 2.12 | 90 |
|  | Subscale ½ mean | -1.47 | 2.21 | 86 | -5.27 | 28.23 | 0 |
| 0.875 (12) | Subscale mean | -0.75 | 0.72 | 99 | -2.11 | 5.14 | 68 |
|  | Subscale ½ mean | -1.45 | 2.16 | 88 | -5.24 | 28.00 | 0 |
| 0.929 (13) | Subscale mean | -1.16 | 1.47 | 94 | -3.78 | 15.13 | 9 |
|  | Subscale ½ mean | -1.44 | 2.16 | 85 | -5.25 | 28.06 | 0 |
